# High-resolution bacterial 16S rRNA gene profile meta-analysis and biofilm status reveal common colorectal cancer consortia

**DOI:** 10.1038/s41522-017-0040-3

**Published:** 2017-11-29

**Authors:** Julia L. Drewes, James R. White, Christine M. Dejea, Payam Fathi, Thevambiga Iyadorai, Jamuna Vadivelu, April C. Roslani, Elizabeth C. Wick, Emmanuel F. Mongodin, Mun Fai Loke, Kumar Thulasi, Han Ming Gan, Khean Lee Goh, Hoong Yin Chong, Sandip Kumar, Jane W. Wanyiri, Cynthia L. Sears

**Affiliations:** 1 Johns Hopkins University School of Medicine, 1550 Orleans St., Baltimore, MD 21231 USA; 2 Resphera Biosciences, 1529 Lancaster St., Baltimore, MD 21231 USA; 3 University of Malaya Faculty of Medicine, 50603 Kuala Lumpur, Malaysia; 4 University of Maryland School of Medicine, Institute for Genome Sciences, 801W. Baltimore St., Baltimore, MD 21201 USA; 5 Monash University Malaysia, School of Science, 47500 Bandar Sunway, Selangor, Darul Ehsan Malaysia

## Abstract

Colorectal cancer (CRC) remains the third most common cancer worldwide, with a growing incidence among young adults. Multiple studies have presented associations between the gut microbiome and CRC, suggesting a link with cancer risk. Although CRC microbiome studies continue to profile larger patient cohorts with increasingly economical and rapid DNA sequencing platforms, few common associations with CRC have been identified, in part due to limitations in taxonomic resolution and differences in analysis methodologies. Complementing these taxonomic studies is the newly recognized phenomenon that bacterial organization into biofilm structures in the mucus layer of the gut is a consistent feature of right-sided (proximal), but not left-sided (distal) colorectal cancer. In the present study, we performed 16S rRNA gene amplicon sequencing and biofilm quantification in a new cohort of patients from Malaysia, followed by a meta-analysis of eleven additional publicly available data sets on stool and tissue-based CRC microbiota using Resphera Insight, a high-resolution analytical tool for species-level characterization. Results from the Malaysian cohort and the expanded meta-analysis confirm that CRC tissues are enriched for invasive biofilms (particularly on right-sided tumors), a symbiont with capacity for tumorigenesis (*Bacteroides fragilis*), and oral pathogens including *Fusobacterium nucleatum*, *Parvimonas micra*, and *Peptostreptococcus stomatis*. Considered in aggregate, species from the Human Oral Microbiome Database are highly enriched in CRC. Although no detected microbial feature was universally present, their substantial overlap and combined prevalence supports a role for the gut microbiota in a significant percentage (>80%) of CRC cases.

## Bowel cancer: Spotlight of suspicion on bacteria

Many studies have found a link between gut microbes and bowel cancer, the third most common cancer worldwide. The details of the association, however, have remained elusive. Researchers in the USA and Malaysia, led by Dr. Cynthia Sears at John Hopkins School of Medicine in Maryland, examined mucosal biofilm status by fluorescence microscopy and performed a meta-analysis of bacterial genetic associations in stool and colon tissues to clarify the connection. They found that bowel cancers were enriched in invasive bacterial biofilms as well as several specific gut and oral species, including one - *Fusobacterium nucleatum* - known to promote tumorigenesis in mouse models. Analyzing gut microbial populations might help assess bowel cancer risk. Further research is needed, however, to determine if these  bacteria directly contribute to disease causality.

## Introduction

While numerous environmental risk factors for colorectal cancer (CRC) were established decades ago,^1^ only in recent years have we begun to appreciate the role of the gut microbiome in CRC. Over a dozen studies have now established that CRC is associated with some form of microbial dysbiosis in terms of taxonomic composition and/or mucosal structural organization (e.g., biofilms), as detected either via stool or tumor tissue.^2–16^ However, these CRC microbiome profiling studies have yielded inconsistent findings, likely due to differences in study design and analysis methodologies. Furthermore, the relative contribution of mucosal vs. luminal (i.e., stool) populations, known to differ,^17^ is uncertain. In the present study, we analyzed the taxonomic composition and biofilm status of both previous and new cohorts in order to present a more robust depiction of the contribution of the microbiota to CRC.

## Results

### Confirmation of the biofilm-spatial relationship in CRC

**Fig. 1:**
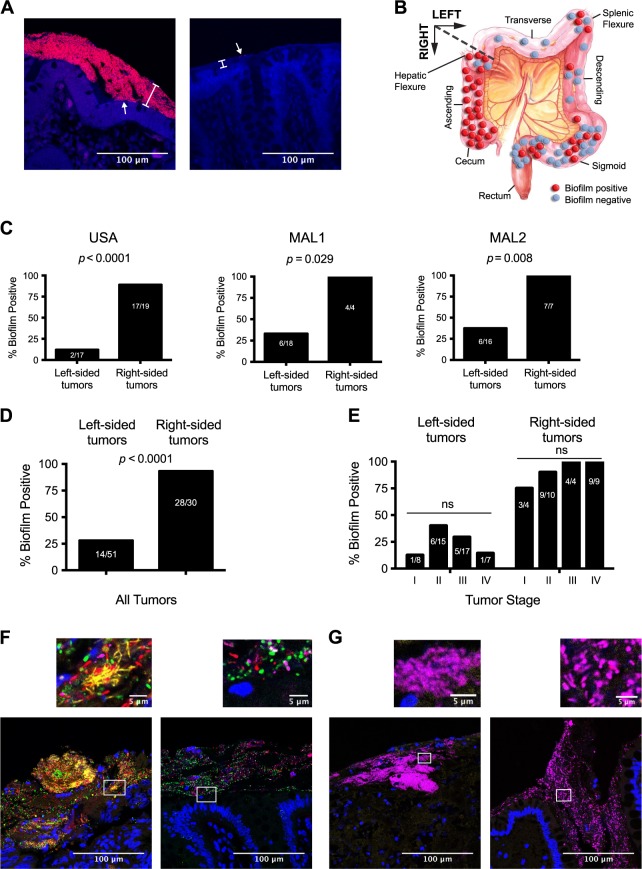
Meta-analysis of biofilm prevalence in CRC. **a** Microbial biofilms in Carnoy’s-fixed tissue were detected by FISH with the universal 16S rRNA gene probe Eub338 (stained in red) and the nucleic acid stain DAPI (blue). White brackets denote the mucus layer, visible via autofluorescence of the tissue. Left panel: a biofilm-positive paired normal tissue from the right colon of a CRC patient with abundant bacteria adjacent to the epithelium (arrow). Right panel: a biofilm-negative paired normal tissue from the left colon of a CRC patient with minimal bacteria (arrow) present only in the outer edge of the mucus. Scale bars represent 100 μm. **b** The locations of tumors from USA, MAL1, and MAL2 are overlaid on a diagram of the colon. **c** Percentage of biofilm-positive right and left tumors for the three cohorts. All statistics shown are Fisher’s exact tests. **d** Total percentage of biofilm-positive right- and left-sided tumors from all three cohorts is depicted with a Fisher’s exact test *p*-value. **e** Left-sided and right-sided tumors separated according to tumor stage and biofilm status. Multivariate logistic regression analysis was performed after controlling for tumor side. **f**, **g** Biofilm-positive samples were stained with DAPI (blue) and probes against four bacterial membership groups: *Fusobacterium* (yellow), Bacteroidetes (green), Lachnospiraceae (red), and Proteobacteria (magenta). Scale bars in large images represent 100 μm; scale bars in smaller, inset images represent 5 μm. **f** Representative polymicrobial biofilm from a tumor (left panel) and its paired normal tissue (right panel), with blooms of *Fusobacterium* in yellow visible only in the tumor. **g** Representative Proteobacteria-dominant tumor (left panel) and its paired normal tissue (right panel)

We first sought to validate previous findings that invasive biofilms are a feature of right-sided (proximal) CRC, originally described in a USA cohort (USA, *N* = 36) and a Malaysian cohort (MAL1, *N* = 22).^16^ Biofilms form when bacteria aggregate on a surface (biological or inert) and become encased in a polymeric matrix.^18,19^ Similar to other in vivo biofilms including oral biofilms (dental plaque) and *Pseudomonas aeruginosa* biofilms in cystic fibrosis patients, the colonic biofilm matrix may consist of both host mucus and bacteria-produced extrapolymeric substrates.^20,21^ The invasion and attachment of biofilms to the colonic epithelium and inner mucus layer can be visualized by a number of different microscopy-based methodologies including fluorescence in situ hybridization (FISH) staining with oligonucleotide fluorescent probes, electron microscopy, or acridine orange staining.^22,23^ Tumor and paired normal samples from a new cohort of 23 patients in Malaysia who underwent surgical resection (described hereafter as MAL2; see Supplementary Table S1 for metadata) were thus screened by FISH with the Eub338 universal 16S rRNA gene probe, using slides from tissue blocks fixed in Carnoy’s to preserve the mucus layer. Paired normal samples were taken from a non-cancerous region of the resected colorectal sample as far as possible from the tumor itself (Supplementary Fig. S1a). Representative biofilm-positive (right-sided) and biofilm-negative (left-sided) tissues from MAL2 are shown in Fig. 1a. A total of 6/16 left-sided tumors from MAL2 were biofilm positive (37.5%), while all seven right-sided tumors were biofilm positive (100%). Biofilm results for MAL2 were then combined with the previously published USA and MAL1 cohorts.^16^ The spatial distribution of tumors and their biofilm status for all three cohorts are depicted in Fig. 1b. Individual study tumor maps are shown in Supplementary Fig. S1b. Each study individually had a significantly higher prevalence of biofilms on right-sided tumors by Fisher’s exact test (Fig. 1c; USA *p* < 0.0001; MAL1 *p* = 0.029; MAL2 *p* = 0.008), as did the combined analysis of all three studies (Fig. 1d, *p* < 0.0001). However, the Malaysian cohorts MAL1 and MAL2 had approximately a 3-fold higher prevalence of biofilms on left-sided tumors than the USA cohort (33% for MAL1, 38% for MAL2, and 12% for USA), which may reflect differences in diet/lifestyle, genetics, or the native microbial environment of the two countries. As previously reported for the USA cohort, the biofilm status of paired normal tissues from MAL1 and MAL2 largely matched the biofilm status of the tumors from the same individual and therefore were called concordant pairs; only 2/21 MAL1 and 3/23 MAL2 patients had discordant biofilm scores between their tumor and normal tissues, all 5 of which were cases in which the tumor was biofilm positive but the paired normal tissue was negative. Multivariate logistic regression analysis revealed that tumor stage [*N* = 74 cancers, excluding surgical polyps (6 from USA; 1 from MAL1)] was not significantly associated with biofilm status after controlling for tumor side (Fig. 1e, right tumors *p* > 0.48 for all stages; left tumors *p* > 0.19 for all stages).

The concordant tumor-normal biofilm-positive pairs from MAL1 (nine pairs) and MAL2 (ten pairs) were subsequently examined by multi-probe FISH with probes against four taxonomic groups that were previously found to be the dominant bacterial members of biofilms in the USA cohort: phylum Bacteroidetes (including *Bacteroides*, *Parabacteroides*, and *Prevotella*), genus *Fusobacterium*, family Lachnospiraceae, and the classes Gamma- and Betaproteobacteria. As with the USA cohort, the Malaysian biofilms tended to be polymicrobial, with an abundance of Lachnospiraceae, Bacteroidetes, and Proteobacteria in both tumor and normal tissues from 17/19 patients. *Fusobacterium* was also detectable in almost all (16/17) tumors with polymicrobial biofilms, ranging from sparse populations (less than 5 bacteria visible in a 200 μm × 200 μm field of view) in the majority of tumors to dense blooms in 4/17 of the biofilm-positive tumors (Fig. 1f). In contrast, paired normal tissues were largely devoid of *Fusobacterium* (Fig. 1f). Sequencing analyses (see subsequent sections) from frozen tissues of the same patients revealed that *F. nucleatum* was often the most abundant fusobacterial species detected in these tissues. The remaining two patients’ biofilms consisted exclusively of Proteobacteria in both the tumor and paired normal tissue (representative tumor/normal staining shown in Fig. 1g). Thus, tumors and paired normal tissues were largely concordant with respect to both biofilm status and biofilm composition with the exception of *Fusobacterium*, which was largely present only in tumor biofilms.

### Microbial taxa and functions associated with biofilm status

To complement the results of biofilm quantification, we next sought to identify microbial members associated with biofilm-positive status. Traditional analyses of 16S rRNA gene sequencing data have been limited to genus-level identification. To enhance our taxonomic resolution, 16S rRNA amplicon sequence data sets from USA, MAL1, and MAL2 cohorts were analyzed using Resphera Insight, a high-resolution methodology for species-level characterization (see Methods).^24–26^ We then assessed significant enrichment or depletion of taxa in biofilm-positive vs. biofilm-negative samples.

**Fig. 2:**
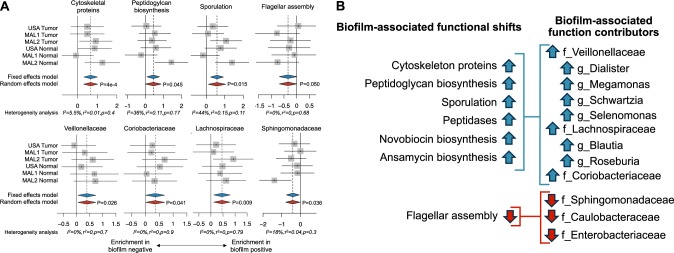
Microbial associations with biofilm status. Frozen tissue from USA, MAL1, and MAL2 cohorts were characterized by 16S rRNA amplicon sequencing. **a** Top panel: biofilm positive CRC and normal flanking tissues demonstrated several functional shifts in the bacterial composition, including increases in gene content associated with cytoskeletal proteins, peptidoglycan biosynthesis, sporulation, and flagellar assembly based on PICRUSt analysis. Bottom panel: the bacterial families Veillonellaceae, Coriobacteriaceae, and Lachnospiraceae were enriched in biofilm-positive samples, while Sphingomonadaceae was enriched in biofilm-negative samples. Random-effects models with 95% CI above or below 0 (red diamonds) were considered statistically significant. Hedge’s *g* difference statistic is shown on the *X* axes. The fixed effects model assumes there exists a single effect size shared by all included studies, while the random effects model allows for variation in the effect size from study to study. Heterogeneity analysis includes estimates of *I*^2^ (percentage of variation reflecting true heterogeneity), *τ*^2^ (random-effects between study variance), and *p*-value from Cochran’s *Q* test for heterogeneity. **b** The functional alterations due to biofilm status were linked to multiple, differentially abundant families (f) and genera (g) in the biofilms

A limited number of specific species were identified as differentially abundant between biofilm-positive and biofilm-negative tissues (Supplementary Fig. S2). Of the four species, one was additionally found to be enriched in right-sided vs. left-sided samples (*Clostridium ramosum*), indicating a potential association with geographical location rather than strictly biofilm status (Supplementary Fig. S3). Despite the limited number of differences at the species level between biofilm-positive and biofilm-negative tissues, a meta-analysis of functional in silico predictions by PICRUSt revealed several biofilm-associated functional shifts, including an increase in gene content attributed to cytoskeletal proteins, peptidoglycan biosynthesis, sporulation, peptidases, novobiocin biosynthesis, and ansamycin biosynthesis, as well as a reduction in flagellar assembly genes (Fig. 2a). Examination of taxa contributing most strongly to these functional associations highlighted multiple, differentially abundant families including significant enrichment of Veillonellaceae, Lachnospiraceae, and Coriobacteriaceae that corresponded to the enhanced functions in biofilm-positive samples, whereas a significant decrease in relative abundance of Sphingomonadaceae and a trend towards lower levels of Caulobacteraceae and Enterobacteriaceae contributed to reductions in flagellar assembly (Fig. 2b).

### Meta-analysis of microbial associations with CRC status independent of biofilms

**Fig. 3:**
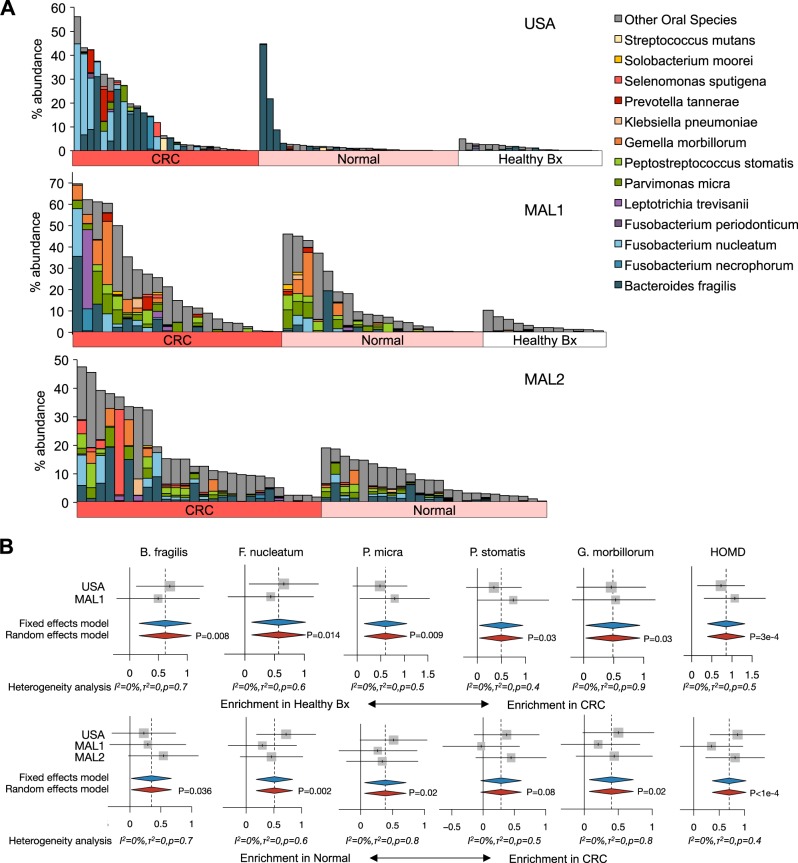
Associations of *B. fragilis* and human oral microbiota with CRC status for USA, MAL1, and MAL2 cohorts. Microbiome profiling with 16S rRNA gene sequencing was applied to tumor (CRC) and paired normal tissues (Normal) from CRC patients for all three cohorts, as well as healthy biopsies (Healthy Bx) for USA and MAL1. **a** Bar charts of microbial sequence relative abundance for *B. fragilis* and bacteria from the Human Oral Microbiome Database (HOMD) from the three 16S rRNA amplicon data sets: USA (top panel), MAL1 (middle panel), and MAL2 (bottom panel). Each vertical bar represents an individual patient. **b** *B. fragilis* as well as the oral pathogens *F. nucleatum*, *P. micra*, *P. stomatis, G. morbillorum*, and the overall consortia associated with the Human Oral Microbiome Database (HOMD) were all found to be significantly enriched in tumor specimens compared to biopsies from healthy patients without cancer (top panel), and compared to normal flanking tissue (bottom panel). Random-effects models with 95% CI above or below 0 (red diamonds) were considered statistically significant. Hedge’s *g* difference statistic is shown on the *X* axes. The fixed effects model assumes there exists a single effect size shared by all included studies, while the random effects model allows for variation in the effect size from study to study. Heterogeneity analysis includes estimates of *I*^2^ (percentage of variation reflecting true heterogeneity), *τ*^2^ (random-effects between study variance), and *p*-value from Cochran’s *Q* test for heterogeneity

We next examined bacterial composition independently of biofilm status in the USA, MAL1, and MAL2 cohorts. For USA and MAL1, CRC samples were compared to both paired normal as well as healthy biopsies, whereas for MAL2 only CRC and paired normal tissues were examined (Fig. 3a). We observed a significant enrichment of the human gut commensal *Bacteroides fragilis* in CRC compared to both normal flanking tissue and healthy biopsies (Fig. 3b). Additionally, four species known to be oral pathogens were also significantly enriched in CRC tissue: *Fusobacterium nucleatum*, *Parvimonas micra*, *Peptostreptococcus stomatis*, and *Gemella morbillorum*. Given this strong association between oral pathogens and CRC, we subsequently looked for enrichment of total oral bacteria from the Human Oral Microbiome Database (HOMD), a curated list of bacteria derived from 16S rRNA gene sequencing of healthy and diseased oral samples from patients (see Supplementary Table S2 for list of species).^27^ This analysis revealed a robust, significant enrichment of total HOMD bacterial species in CRC tissue compared to both paired normal tissue and healthy biopsies. Enrichment of the species identified above in the tumors was not specific to tumor location; both right-sided tumors and left-sided tumors contributed to the enrichment of *F. nucleatum*, *P. micra*, *P. stomatis*, *G. morbillorum*, and HOMD species (Supplementary Fig. S4). To further validate these initial findings, we chose samples from the MAL1 cohort and performed qPCR for the 16S rRNA gene for *B. fragilis* and *F. nucleatum*. Read counts from the 16S rRNA amplicon sequencing analysis for both *B. fragilis* and *F. nucleatum* significantly correlated with copy numbers from 16S rRNA gene qPCR (Supplementary Fig. S5).

The above analysis was then expanded to include all available 16S rRNA gene sequencing data sets on CRC samples, including two additional studies involving CRC versus healthy biopsy samples,^8,15^ five studies on CRC versus paired normal tissue,^2,3,6,15,28^ and four stool-based studies.^4,5,9,28^ In total, eight data sets were analyzed for differences between CRC and healthy biopsies and/or stool, and eight data sets were analyzed for differences between CRC and paired normal tissue. Table 1 summarizes the country of origin, tissue type (stool vs. tissue biopsy), DNA extraction method, sequencing platform, 16S rRNA gene primers, and original findings of each publication as reported by their respective authors prior to our meta-analysis. Raw data sets were preprocessed using a standardized methodology (see Methods). Collectively, this analysis encompassed populations in North America (U.S., Canada), Europe (France, Germany, Spain) and Asia (China, Vietnam, Malaysia), and represented stools from 481 tumor patients and 271 healthy controls, as well as colon tissues from 379 tumors, 369 paired normal tissues from tumor hosts and 172 biopsies (from 89 patients).

**Table 1:** CRC cohorts included in the expanded 16S rRNA meta-analysis alongside original findings of the authors

Geographical location of cohort	Methodology (16S primer set/Sequencing platform/DNA prep)	Sample size	Enriched in tumor (T) compared to paired normal (N) or healthy biopsy (H)	Reference
Canada and USA	V4/Illumina MiSeq/PowerSoil-htp 96 Well Soil DNA isolation kit	318 tumor stools, 172 healthy stools	T stool vs. H stool g_Porphyromonas g_Peptostreptococcus g_Fusobacterium g_Parvimonas g_Prevotella g_Gemella	Baxter et al. 2016^5^
USA	V5-V6/Illumina MiSeq/Qiagen All-prep kit with sonication in Qiazol lysis solution	44 T/N pairs	T vs. N g_Fusobacterium, g_Candidatus, g_Portiera, g_Providencia	Burns et al. 2015^2^
USA	V3-V5/454 pyrosequencing/QIAamp DNA Stool Mini kit with pressure lysis	30 T/N pairs with 2 matched stools; 22 biopsies from 11 healthy patients	N/A; original analysis focused on bacteria associated with biofilm status	Dejea et al. 2014^16^
China	V1-V2/454 pyrosequencing/QIAamp DNA Mini kit with bead-beating	8 T/N pairs, 10 additional adenomas, 77 biopsies from 11 healthy patients	T (carcinoma) vs. N g_Roseburia	Geng et al. 2013,^6^ Geng et al. 2014,^7^ Zhang et al. 2013^8^
Spain, Vietnam, and USA	V3-V5/454 pyrosequencing/standard tissue DNA extraction performed at a core facility, exact method unknown	95 T/N pairs	T vs. N g_Fusobacterium s_F.nucleatum s_F.necrophorum s_F.mortiferum	Kostic et al. 2012^3^
China	RT-PCR and 454 pyrosequencing/QIAamp DNA Mini kit with additional bead-beating and lysozyme digestion	99 T/N pairs, 61 healthy controls	T (carcinoma) vs. N and H s_B.fragilis g_Gemella g_Parvimonas g_Peptostreptococcus g_Granulicatella	Nakatsu et al. 2015^15^
France	V3-V4/454 pyrosequencing/NA	6 tumor stools, 6 healthy stools	T stool vs. H stool g_Bacteroides + g_Prevotella	Sobhani et al. 2011^4^
Malaysia 1	V3-V4 / Illumina MiSeq/Zymo Fecal DNA Kit with bead-beating and lysozyme step	22 T/N pairs; 12 biopsies from 6 healthy patients	This study	This study
Malaysia 2	V3-V4/Illumina MiSeq/MasterPure DNA Purification kit	23 T/N pairs + 3 T/N paired biological replicates from MAL1	This study	This study
Canada and USA	V4 / Illumina MiSeq / PowerSoil-htp 96 Well Soil DNA isolation kit	60 tumor stools, 30 healthy stools	T (carcinoma) stool vs. H stool g_Fusobacterium g_Porphyromonas	Zackular et al. 2014^9^
France	V4 / Illumina MiSeq / GNOME DNA Isolation Kit with modifications	95 tumor stools, 61 healthy stools	T stool vs. H stool s_F.nucleatum s_P.asaccharolytica s_C.symbiosum s_B.fragilis s_E.rectale s_E.ventriosum s_E.eligens s_S.salivarius	Zeller et al. 2014^28^
Germany	V4 / Illumina MiSeq / GNOME DNA Isolation Kit with modifications	48 T/N pairs	T vs. N s_F.nucleatum s_E.ventriosum s_E.eligens s_S.salivarius	Zeller et al. 2014^28^

*g* genus, *s* species

**Fig. 4:**
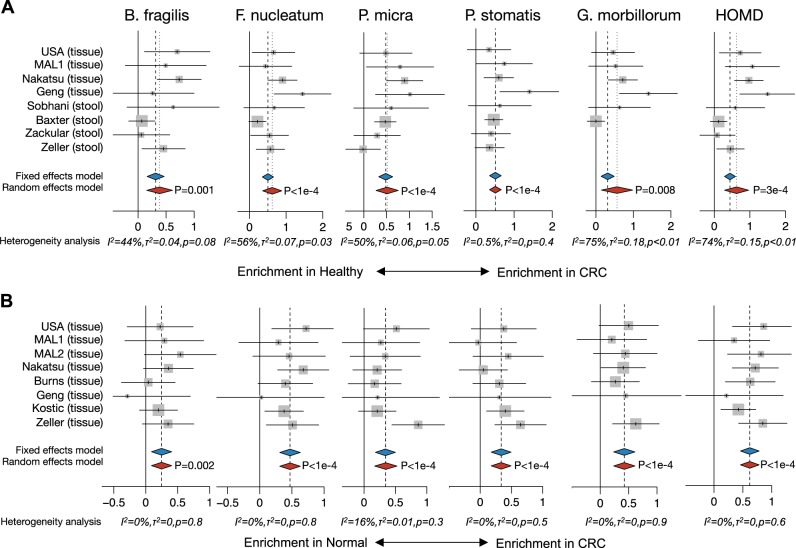
Expanded differential relative abundance meta-analysis supports species-level findings. A meta-analysis of **a** CRC tissue or stool vs. healthy biopsy or stool and **b** CRC vs. flanking normal tissues supports the enrichment of *B. fragilis* and oral microbes in CRC. Random-effects models with 95% CI above or below 0 (red diamonds) were considered statistically significant. Hedge’s *g* difference statistic is shown on the *X* axes. The fixed effects model assumes there exists a single effect size shared by all included studies, while the random effects model allows for variation in the effect size from study to study. Heterogeneity analysis includes estimates of *I*^2^ (percentage of variation reflecting true heterogeneity), *τ*^2^ (random-effects between study variance), and *p*-value from Cochran’s *Q* test for heterogeneity

Enrichment of specific species in the initial USA/MAL1/MAL2 cohort analysis was validated in this expanded analysis: *B. fragilis*, *F. nucleatum*, *P. micra*, *P. stomatis*, *G. morbillorum*, and the total oral microbiome from HOMD (Fig. 4). The statistical significance for each individual microbe from HOMD is provided in Supplementary Table S2. We found these enrichments to be robust to variations in normalization strategy and confidence in species-level assignment (Supplementary Fig. S6). The stool studies also supported these findings, although the signal was weaker in comparison to the robust differences seen in tissues (Fig. 4). Although others have reported an enrichment of fusobacterial species and the enterotoxigenic strain of *B. fragilis* in late-stage tumors,^29,30^ we did not observe any consistent changes with respect to *F. nucleatum*, *B. fragilis*, HOMD, or combinations of these species and tumor stage (Supplementary Fig. S7). Conversely, several *Bacteroides* species (*B.vulgatus*, *B. dorei*, and *B. stercoris*) as well as *Faecalibacterium prausnitzii* were consistently depleted in CRC compared to healthy biopsies and paired normal tissues (Supplementary Fig. S8).

### Not all fusobacterial species are enriched in CRC

**Fig. 5:**
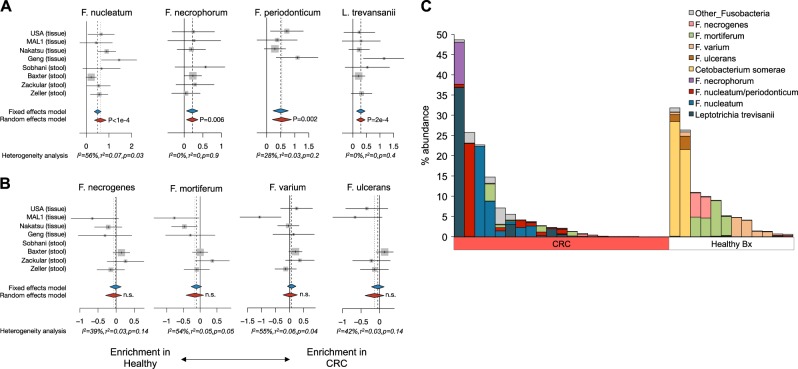
Not all fusobacterial species are associated with CRC. **a** In addition to *F. nucleatum*, we detected significant enrichment of *F. necrophorum*, *F. periodonticum*, and *L. trevansanii* in CRC tissue or stool compared to healthy biopsies or stool. Hedge’s *g* difference statistic is shown on the *X* axes. The *F. periodonticum* forest plot includes ambiguous assignments (see Methods). **b** Other fusobacterial species were not differentially abundant between CRC and healthy patients. The fixed effects model assumes there exists a single effect size shared by all included studies, while the random effects model allows for variation in the effect size from study to study. Heterogeneity analysis includes estimates of *I*^2^ (percentage of variation reflecting true heterogeneity), *τ*^2^ (random-effects between study variance), and *p*-value from Cochran’s *Q* test for heterogeneity. **c** Bar chart of all detected species within the fusobacterial phylum and their total sequence relative abundances in the MAL1 cohort. Each vertical bar represents an individual patient. Several healthy Malaysian patients in the MAL1 cohort harbored high levels of fusobacterial species not associated with CRC

The expanded meta-analysis also uncovered significant enrichment of additional fusobacterial species *F. necrophorum*, *F. periodonticum*, and *L. trevansanii* in CRC samples compared to healthy biopsies (Fig. 5a). However, there were several other species within the fusobacteria phylum that were not enriched in CRC and were in some cases more highly abundant in the healthy tissues. Fusobacteria not associated with CRC included *F. necrogenes*, *F. mortiferum*, *F. varium*, and *F. ulcerans* (Fig. 5b). Additionally, two healthy biopsy patients from Malaysia (MAL1) harbored very high levels of the Fusobacteriaceae member *Cetobacterium somerae* (>20% relative abundance of all reads; Fig. 5c). Such data highlight the importance of detection down to the species level, as only a subset of the genus *Fusobacterium* was associated with CRC and an analysis limited to genera would therefore have resulted in a less robust or even non-significant signal. Moreover, the highly abundant intestinal commensal *Faecalibacterium prausnitzii* was originally classified as *Fusobacterium prausnitzii* until 2002;^31^ had this species not been reclassified, it would have strongly confounded genus and higher-level signals associated with CRC.

### Compilation of all microbial features of CRC

**Fig. 6:**
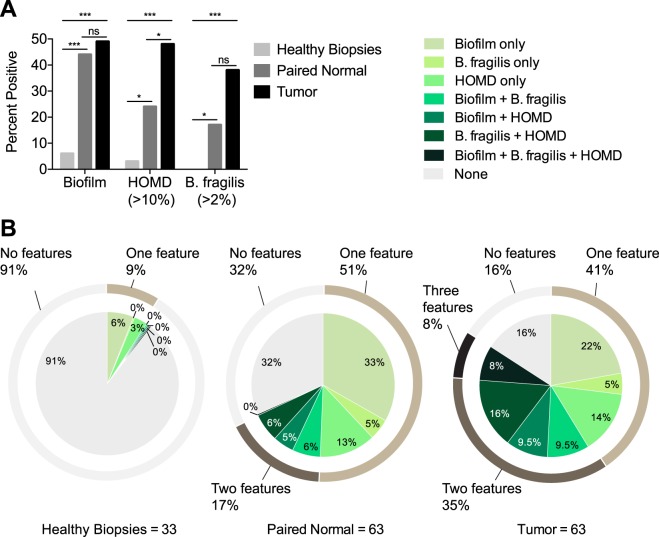
Combined prevalence of CRC-associated microbial features in USA, MAL1, and MAL2 cohorts. Positive status for *B. fragilis* and the human oral microbes from the HOMD database were defined as a relative abundance >2 and >10% of all 16S rRNA gene sequences, respectively. **a** Percentage of healthy biopsies, paired normal, and tumor tissues that were positive for each microbial feature (biofilms, HOMD, and *B. fragilis*). Statistics shown are Fisher’s exact test. *p < 0.01, ***p < 0.001. **b** Percentage and overlap of healthy biopsies, paired normal, and tumor tissues harboring one, two, three or no microbial features

Finally, we assessed the combined prevalence of biofilms, *B. fragilis*, and oral microbes from HOMD in order to determine the percentage of CRC tumors with at least one feature of microbial dysbiosis. We selected samples for which data on all three microbial features were available (i.e., samples from the USA, MAL1 and MAL2 cohorts). We defined enrichment of *B. fragilis* as tumors with *B. fragilis* relative abundance >2% of overall sequences, and enrichment of HOMD as tumors having HOMD bacteria present at >10% of overall sequences; these cut off points excluded >90% of healthy biopsies. Tumors harbored the highest percentages of all three features: 49% were biofilm-positive, 48% were HOMD positive, and 38% were *B. fragilis* positive (Fig. 6a). While a similar percentage of paired normals were biofilm positive (44%, *p* = 0.721 by Fisher’s exact test compared to tumors), the prevalence of HOMD and *B. fragilis* in paired normal tissues was approximately half of that found in tumors, significantly so for HOMD (HOMD: 24 vs. 48%, *p* = 0.009; *B. fragilis*: 17 vs. 38%, *p* = 0.254, respectively; Fig. 6a). Only 2/11 paired normal tissues that were positive for *B. fragilis* had tumors that were below the cut-off value, while similarly 3/15 paired normal tissues that were positive for HOMD had tumors below the HOMD cut-off value. Of the 33 healthy biopsies randomly chosen for sequencing, representing left and right biopsies from 16 patients and one biopsy from the right side of a 17th patient, only 6% were biofilm positive (2/33), with both biofilms occurring on right-sided biopsies from the MAL1 cohort (Supplementary Table S1). Three percent of the biopsies were enriched for HOMD organisms, and 0% were enriched for *B. fragilis*. These percentages were significantly lower than the percentages observed for paired normals and tumors for each feature by Fisher’s exact test (healthy biopsy vs. paired normal: *p* < 0.0001 for biofilms, *p* < 0.01 for HOMD, *p* < 0.01 for *B. fragilis*; healthy biopsy vs. tumor: *p* < 0.0001 for all three features).

In addition to having the highest percentages of each individual microbial feature above, an abundance of tumors (27/63 or 43% total) harbored more than one feature (41% single, 35% two, and 8% three features) (Fig. 6b). When tumors were analyzed according to their anatomical location, right-sided tumors, not unexpectedly, displayed more biofilm-related categories, while the predominant phenotype of left-sided tumors reflected the co-occurrence of *B. fragilis* and HOMD (Supplementary Fig. S9). In contrast to the tumors, paired normal tissue contained mostly single features (51% single, 17% two, and 0% three features), with the majority of those harboring more than one feature also having tumors with more than one feature (8/11). The healthy biopsies contained only single features (6% biofilm only, 3% HOMD only) and were otherwise absent of any dysbiosis (91% no features). Overall, 84% of tumors harbored at least one measure of microbial dysbiosis associated with CRC status as defined in our meta-analysis, compared to 68% of paired normal tissues and only 9% of healthy biopsies.

## Discussion

Overall, our meta-analysis demonstrates that the vast majority (>80%) of CRC cases contain aberrant microbial signatures indicative of dysbiosis. Our high-resolution 16S rRNA gene meta-analysis captured species-level taxonomic assignments, allowing for the most detailed map to date of the microbial dysbiosis in CRC. These data support multiple avenues by which bacteria may promote carcinogenesis, including enrichment of a symbiote with enterotoxigenic capabilities (*B. fragilis*), the emerging role of oral microbes as potentially hostile guests in the gut, and the establishment of polymicrobial, procarcinogenic biofilms.^16,32,33^ Additionally, while specific organisms, such as *B. fragilis* and *F. nucleatum* have been implicated by individual studies using alternative methods such as metagenomic sequencing or qPCR, the present meta-analysis pipeline enabled consistent detection of these species by 16S rRNA gene sequencing—often in cases where the original authors’ findings were restricted to genus-level findings—regardless of the sequencing platform, 16S rRNA gene primer set, or DNA extraction method used. Importantly, the microbial features detected in our study were not mutually exclusive and in fact were often found to co-occur in patients in our meta-analysis. However, whether these microbial features are required for the initiation or progression of tumorigenesis or whether they are merely bystanders remains to be elucidated.

*B. fragilis* is a well-studied human gut symbiont, making its enrichment associated with CRC status potentially surprising. However, enterotoxigenic strains of *B. fragilis* (ETBF) harboring the *B. fragilis* toxin (BFT) have long been associated with diarrheal disease and, potentially, inflammatory bowel disease and CRC.^30,34^ Mouse and in vitro studies have shown that BFT likely promotes tumorigenesis by inducing (1) DNA damage through enhanced spermine oxidase and resulting ROS activity,^35^ and (2) cleavage and subsequent degradation of the tumor suppressor E-cadherin, resulting in increased permeability of the colonic epithelium, enhanced Wnt/β-catenin signaling, and enhanced cellular proliferation.^36,37^ In mouse models, inoculation of ETBF into *Apc*^Min/+^ mice accelerates tumorigenesis in an IL-17-dependent manner.^38^ In patients, *bft* has been found to be present more frequently in CRC versus controls in both the colon mucosa (>85 vs. 53%)^30^ and in the stool (38 vs. 12%, and 27 vs. 10%).^39,40^ Thus, the association between enrichment of *B. fragilis* and CRC observed in our meta-analysis may in fact reflect enrichment of ETBF strains of *B. fragilis* in the gut. Importantly, the enrichment of *B. fragilis* was paralleled by depletion of several other *Bacteroides* members, which is consistent with reports by others of an overall loss of phylum Bacteroidetes^3^ and more specifically the genus *Bacteroides*.^9,12^ The selective enrichment of *B. fragilis* may therefore suggest a growth advantage of *B. fragilis* over other Bacteroides species during tumorigenesis, and further highlights the importance of species-level resolution.

Our meta-analysis also revealed a robust enrichment of *F. nucleatum* and other oral species in CRC compared to both paired normal and healthy biopsy tissues. These data are consistent with a recent meta-analysis by Shah et al. in stool samples in which *Parvimonas micra* and two separate unclassified *Fusobacterium* were reported to be enriched in stools from CRC patients compared to healthy controls.^41^ However, that study was unable to identify *F. nucleatum* specifically, likely due to the signal being weaker in stool compared to tissue and/or the enhanced resolution of our meta-analysis pipeline compared to the strain select tool SS-UP used in that study. *F. nucleatum* has gained increasing notoriety in recent years as a potential pathogen in a number of clinical diseases including gastro–intestinal disorders, cardiovascular disease, adverse pregnancy outcomes, respiratory tract infections, and oropharyngeal infections, where it was first discovered.^42^ Mechanistically, *F. nucleatum* is a ubiquitous oral bacterium that is both highly adherent and invasive, a property attributed to virulence proteins including the adhesion and invasion protein, FadA, and the galactose-inhibitable adhesion protein Fap2.^43–45^ More recently, secreted or surface FadA was found to bind to E-cadherin and induce Wnt/β-catenin signaling in the colons of mice, where *F. nucleatum* accelerated tumorigenesis.^46,47^ Immune mechanisms including myeloid cell infiltration, the balance between FOXP3^hi^ and FOXP3^lo^ Treg populations, and checkpoint molecule (TIGIT) blockade may modulate *F. nucleatum*-associated carcinogenesis.^47–50^

However, *Fusobacterium* are enriched in only a subset of tumors and are present at a much lower abundance in paired normal tissue.^3,13,46,51–53^ Similarly, enrichment of *Fusobacterium* in biofilms occurs in only a subset of tumors and is often undetectable in paired normal tissues,^16^ findings that our additional cohort of Malaysian patients and meta-analyses upheld. As most carcinogenic mechanisms are predicted to be elevated in adjacent normal tissue as well as the tumor itself, in line with the cancer hypothesis that it takes years or even decades for cancer to develop within normal colon tissue, these data question whether *F. nucleatum* is an important initiator of carcinogenesis. Instead, *F. nucleatum* may be uniquely suited to grow and contribute to tumor progression in an established tumor microenvironment, due to the microbe’s highly adherent and invasive nature that could exploit a compromised colonic epithelial cell (CEC) layer. Further, as Kostic et al. have proposed, the asaccharolytic metabolism of *F. nucleatum* would not compete for glucose consumption by the tumor, and the anaerobic nature of *F. nucleatum* would enable it to tolerate the hypoxic tumor environment.^47^ *F. nucleatum* may even provide a growth advantage for the tumor, due to the bacteria’s ability to inhibit NK cell-mediated tumor cell death via binding of the bacterial protein Fap2 to one of the NK cell inhibitory receptors, TIGIT.^50^ The procarcinogenic capability of *F. nucleatum* in the gut may be highly strain-dependent, as some strains have been resistant to colonization in mouse gut and required daily inoculation^47^ while others have been found to colonize readily.^49^

*F. nucleatum* is frequently found to co-occur with other oral pathogens in CRC^53^ and can coaggregate with members of all genera in vitro.^54^ As such, *F. nucleatum* may be a bridging organism that assists in the colonization of other microbes through its myriad adhesins, which allow other microbes to adhere to and interact with *F. nucleatum*.^42,55^ Our meta-analysis revealed consistent elevations in not only *F. nucleatum* but also several other oral pathogens (e.g., *P. micra* and *P. stomatis*) in CRC compared to paired normal tissue and healthy biopsy controls. A variety of other oral microbes were frequently detected as well, including several Fusobacteriaceae that were enriched in normal flanking tissues compared to CRC from Malaysian patients. Flynn et al. have elegantly proposed a polymicrobial model in which several oral pathogens, reliant on each other for survival, may establish a niche in the gut that may contribute to tumorigenesis due to similarities between the gut and the oral environment such as similar pH and propensity to form biofilms.^33^ Initiation or progression of tumorigenesis becomes beneficial to these bacteria because of increased nutrients from local inflammatory responses. These organisms include *Fusobacterium*, *Parvimonas*, and *Peptostreptococcus* species found to be enriched in CRC in our meta-analysis, as well as *Porphyromonas*, *Prevotella*, and *Gemella* species enriched in other studies.^5,9,15^

While it is tempting to speculate that biofilms from the oral cavity containing *F. nucleatum* seed the gut biofilms that we have observed in a substantial number of CRC cases, this likely would only account for a subset of biofilm formation events. Sparsely populated (<5 bacteria per 200 μm^2^ area) *Fusobacterium* could be detected in almost all of the biofilms from Malaysia by FISH, but dense fusobacterial aggregates were abundant in only 25% of the tumor biofilms, were infrequently detected in biofilms on paired normal tissue, and were not detected on any healthy biopsies. By sequencing, approximately only one-third of biofilm-positive tumors were enriched for oral consortia derived from the HOMD, while <10% of biofilm-positive paired normal tissues and 0% of biofilm-positive healthy biopsies (*N* = 2) contained HOMD levels of an appreciable relative abundance. These data suggest that oral bacteria such as *F. nucleatum* are not required for biofilm formation in the gut and that therefore the initiating species may be different from that of oral biofilms.

Certainly, further studies on how and why biofilms form in the human gastrointestinal tract are necessary. The data thus far support a role for biofilms in causality of a subset of CRC, particularly right-sided CRC, where nearly 100% of tumors and paired normal tissue have been found to be covered in invasive, polymicrobial biofilms adjacent to the CEC layer. In comparison, a range of 13–35% of healthy screening colonoscopy patients have been reported to harbor colonic biofilms, with equivalent percentages of biofilms on the right and left sides.^16,23^ Notably, the prevalence of biofilms in the healthy biopsies in the present meta-analysis was lower than expected (6%); however, separating these samples by cohort revealed that 2/12 MAL1 biopsies (17%) were biofilm positive, while the 21 USA biopsies chosen for sequencing were part of a larger cohort previously published in which 15/120 biopsies (13%) were biofilm positive.^16^ Clearly there is a need for a much larger study of biofilms in healthy screening colonoscopy individuals to resolve the true estimated prevalence, but the current estimates still point to a much higher rate of biofilms on proximal tumors than on proximal biopsies. The potential procarcinogenic mechanisms of biofilms elucidated thus far include production of the polyamine (N)1, (N)12-diacetylspermine^56^ and induction of a pro-inflammatory response involving enhanced epithelial levels of IL-6, phospho-STAT3, and increased crypt epithelial cell proliferation.^16^ Importantly, one cannot predict biofilm status from sequencing data alone. For biofilm-negative tissues, sequencing likely detected bacteria enmeshed in the loose, permissive outer mucus layer, a distance from the CECs, whereas, for biofilm-positive tissues, bacteria invading the dense, restrictive mucus layer adjacent to the CECs were also detected. Thus, the limited changes in bacterial composition that we observed between biofilm-positive and biofilm-negative samples suggest that the close proximity of the bacteria to the CEC layer and changes in their function within a biofilm (Fig. 2) may be more important to changes in CRC biology than the composition of the biofilm itself.

Overall, our meta-analysis of microbial features in CRC confirms three separate but partially overlapping dysbiotic mechanisms: enrichment of *B. fragilis*, enrichment of oral microbes such as *F. nucleatum*, and a high prevalence of invasive, polymicrobial biofilms. Strengths of the 16S rRNA gene meta-analysis in particular include the fact that these commonalities were found in patients from several different countries in North America, Europe, and Asia, using a wide range of sample preparation techniques and 16S rRNA gene sequencing technologies. The implementation of a high-resolution, consistent analysis methodology greatly improved our ability to detect species-level changes compared to the original authors’ findings, which were largely restricted to genus-level associations (see Fig. 4 vs. Table 1), as well as compared to the recent stool meta-analysis by Shah et al.^41^ Additionally, samples from not only tumors and paired normal tissue but also healthy biopsies and stool samples were included in the analysis. A similar strength of signal was obtained when comparing CRC vs. paired normal (tissues only) or CRC vs. healthy (tissues and stool), although the latter displayed more heterogeneity by *I*^2^ values. However, the stool samples displayed weaker associations than tissues, suggesting that a tissue-based approach would be required for potential biomarker studies involving the identified microbial features. Weaknesses include the fact that the present analysis did not take into account the concepts of mucosal-associated bacterial co-abundance groups^57^ or metacommunities,^15^ which may represent another layer of complexity in the microbiome-CRC hypothesis, although our analysis of total HOMD microbes likely parallels a metacommunity phylotype in the latter study. Furthermore, we did not analyze expression of virulence factors, which are an important nuance of bacterial genetics. Despite these limitations, our data strongly support a clear association between multiple, non-mutually exclusive forms of microbial dysbiosis and a substantial portion of CRC cases (>80%). While we should be cautious in attributing the evolution of carcinogenesis to microbes, defining the subset of patients that may be at risk of microbiome-associated CRC tumorigenesis could be a major turning point in CRC prevention, detection, and treatment. Prospective studies will be necessary to confirm that biofilms, *B. fragilis*, and oral pathogens confer an increased risk of CRC.

## Methods

### Ethical statement

This study was approved by the University of Malaya Medical Centre (UMMC, Kuala Lumpur, Malaysia) Medical Ethics Committee (Ref No. 1066.38) and the Johns Hopkins Institutional Review Board. All samples were obtained in accordance with the Health Insurance Portability and Accountability Act. Blank consent forms for the US and Malaysian cohorts (the latter provided in Malay and English) are available in the Supplementary Information. This research was conducted in accordance to all relevant guidelines and procedures.

### Sample collection

The approach to collection of colon tissues for the USA, MAL1 and MAL2 cohorts has been previously described.^16^ The proximal colon through the hepatic flexure was defined as right colon, and distal to the hepatic flexure as left colon. Samples from MAL1 and MAL2 were maintained as independent cohorts because 16S rRNA amplicon sequencing was performed at different facilities with different methodologies for the two sets of samples. Individuals who had received pre-operative radiation, chemotherapy or had a personal history of CRC or inflammatory bowel disease were excluded. All patients underwent a standard mechanical bowel preparation. Standard pre-operative intravenous, but not oral, antibiotics were administered in all surgical cases. Demographic and histopathology information of the study subjects are summarized in Supplementary Table S1.

Study data were collected and managed using REDCap electronic data capture tools^58^ hosted at Johns Hopkins University.

### FISH analysis of biofilms

FISH was performed on Carnoy’s-fixed, paraffin-embedded tissue sections from the Malaysian patients as previously described, with minor modifications^16^ (see SI Methods). Samples were screened in a randomized, blinded fashion.

### 16S rRNA gene Illumina library generation and sequencing

For the MAL1 cohort, a total of 54 samples were evaluated for sequencing, including paired left-right biopsies from six healthy patients, paired tumor/normal samples from 20 CRC patients, and an additional two unpaired tumors from two CRC patients. One polyp was sequenced but excluded from analysis. DNA was extracted using the ZR Fecal DNA MiniPrep kit (Zymo Research) with modifications (see SI Methods). High-throughput next-generation sequencing of the V3-V4 hypervariable region of the 16S rRNA gene was performed using 319 F (5′-ACTCCTACGGGAGGCAGCAG-3′) and 806 R (5′-GGACTACHVGGGTWTCTAAT-3′) universal primers containing a linker sequence required for Illumina MiSeq 300 bp paired-end sequencing and a 12-bp heterogeneity-spacer index sequence.^59,60^

For the MAL2 cohort, a total of 46 samples, including paired tumor/normal colon tissue samples from 19 CRC patients, two unpaired tumors from CRC patients, and biological replicates of three MAL1 paired tumor/normal samples were evaluated. Removal of the biological replicates from either MAL1 or MAL2 did not influence statistical significance of the analysis (Supplementary Fig. S10). DNA was extracted using the MasterPure DNA Purification Kit (Epicentre/Illumina). The V3-V4 region of the 16S rRNA gene was amplified using S-D-Bact-0341-b-S-17 forward (5′-NNNNCCTACGGGNGGCWGCAG-3′) and S-D-Bact-0785-a-A-21 reverse (5′-GACTACHVGGGTATCTAATCC-3′) primers^61^ designed to include the Illumina-compatible adapters.^62^ SI Methods contain additional details of library generation and sequencing.

### Analysis of all 16S rRNA amplicon sequence data sets

Data sets generated utilizing paired-end sequencing on the Illumina platform were first processed as follows: raw paired-end reads were merged into consensus fragments by FLASH^63^ requiring a minimum 20 bp overlap with 5% maximum mismatch density, and subsequently filtered for quality (targeting error rates <1%) and length (minimum 150 bp) using Trimmomatic^64^ and QIIME.^65,66^ Spurious hits to the PhiX control genome were identified using BLASTN and removed.

Data sets generated utilizing Roche/454 sequencing were first preprocessed as follows: sequences were de-multiplexed using 5′ barcode identifiers and filtered for quality and length in QIIME^65,66^ requiring: (i) trimming of the first 15 bp window with a mean Phred quality score below 24, (ii) a maximum homopolymer run of eight nucleotides, and (iii) a minimum final length of 150 bp. Passing sequences were error-corrected using Acacia with default parameters.^67^

Resulting sequences from all sequencing technologies were then trimmed of their associated primers, evaluated for chimeras with UCLUST (de novo mode),^68^ and screened for human-associated contaminant using Bowtie2^69^ searches of NCBI *Homo sapiens* Annotation Release 106 followed by a BLASTN search against the GreenGenes 16S database (v13.05)^70^ to identify unaligned host-associated sequences. Reads assigned to chloroplast or mitochondrial contaminants by the RDP classifier^71^ with a minimum confidence of 50% were removed.

High-quality 16S sequences were assigned to a high-resolution taxonomic lineage using Resphera Insight (Baltimore, MD).^24–26^ Sequences were also analyzed by PICRUSt^72^ to infer functional content. Species associated with the human oral tract were determined from the HOMD.^27^

Details of Resphera Insight speciation validation, benchmarking studies and statistical analyses are available in SI Methods.

### Code availability

Open source R code used for meta-analyses is available from the authors upon request.

### Data availability

Raw sequences from new cohorts (MAL1 and MAL2) have been deposited in the NCBI SRA repository (BioProject accession no. PRJNA325649 and PRJNA325650). Primary sequencing data are available upon request.

## Electronic supplementary material

Supplementary Methods

Fig S1

Fig S2

Fig S3

Fig S4

Fig S5

Fig S6

Fig S7

Fig S8

Fig S9

Fig S10

Fig S11

Table S1

Table S2

Table S3

## Electronic supplementary material

**Supplementary information** accompanies the paper on the *npj Biofilms and Microbiomes* website (10.1038/s41522-017-0040-3).
