# Author Correction: High-resolution bacterial 16S rRNA gene profile meta-analysis and biofilm status reveal common colorectal cancer consortia

**DOI:** 10.1038/s41522-018-0078-x

**Published:** 2019-01-09

**Authors:** Julia L. Drewes, James R. White, Christine M. Dejea, Payam Fathi, Thevambiga Iyadorai, Jamuna Vadivelu, April C. Roslani, Elizabeth C. Wick, Emmanuel F. Mongodin, Mun Fai Loke, Kumar Thulasi, Han Ming Gan, Khean Lee Goh, Hoong Yin Chong, Sandip Kumar, Jane W. Wanyiri, Cynthia L. Sears

**Affiliations:** 1 Johns Hopkins University School of Medicine, 1550 Orleans St, Baltimore, MD 21231 USA; 2 Resphera Biosciences, 1529 Lancaster St, Baltimore, MD 21231 USA; 3 University of Malaya Faculty of Medicine, 50603 Kuala Lumpur, Malaysia; 4 University of Maryland School of Medicine, Institute for Genome Sciences, 801 W. Baltimore St, Baltimore, MD 21201 USA; 5 Monash University Malaysia, School of Science, 47500 Bandar Sunway, Selangor, Darul Ehsan Malaysia

In this Article, in the section entitled ‘Quantitative real-time PCR’ within the Supplementary Methods, the probe for the *Bacteroides fragilis 16S* real-time PCR reaction was listed incorrectly as 5ʹHEX-AGGGACTGGAAGGCTTTACTGCTTC-3ʹBHQ1. The correct probe for *B. fragilis 16S* should be listed as 5ʹHEX-ACACGTATCCAACCTGCCCTTTACTCG-3ʹBHQ1. The mistake was a result of a copy and paste error with a different primer set targeting a *B. fragilis* toxin gene. All qPCR reactions were performed using the correct probe, and therefore no data were affected.

