# Supplementary material for: High-resolution bacterial 16S rRNA gene profile meta-analysis and biofilm status reveal common colorectal cancer consortia
Source: NPJ Biofilms Microbiomes. 2017 Nov 29;3:34. doi: 10.1038/s41522-017-0040-3 (PMC5707393; doi:10.1038/s41522-017-0040-3)
Supplement: Supplementary file 1 — Supplementary Methods [file 41522_2017_40_MOESM1_ESM.docx]

**Supplementary Information for: High-resolution bacterial 16S rRNA gene profile meta-analysis and biofilm status reveal common colorectal cancer consortia**

Julia L. Drewes^1*^, James R. White^2*^, Christine M. Dejea^1^, Payam Fathi^1^, Thevambiga Iyadorai^3^, Jamuna Vadivelu^3^, April C. Roslani^3^, Elizabeth C. Wick^1^, Emmanuel F. Mongodin^4^, Mun Fai Loke^3^, Kumar Thulasi^3^, Han Ming Gan^5^, Khean Lee Goh^3^, Hoong Yin Chong^3^, Sandip Kumar^3^, Jane W. Wanyiri^1^, Cynthia L. Sears^1^

^1^Johns Hopkins University School of Medicine, 1550 Orleans St., Baltimore, MD 21231 USA

^2^Resphera Biosciences, 1529 Lancaster St., Baltimore, MD 21231, USA

^3^University of Malaya Faculty of Medicine, 50603 Kuala Lumpur, Malaysia

^4^University of Maryland School of Medicine, Institute for Genome Sciences, 801 W. Baltimore St., Baltimore, MD 21201, USA

^5^Monash University Malaysia, School of Science, 47500 Bandar Sunway, Selangor Darul Ehsan, Malaysia

*These authors contributed equally to this work

**Correspondence to:** Cynthia L. Sears; [csears@jhmi.edu](mailto:csears@jhmi.edu)

**SUPPLEMENTARY METHODS**

**Fluorescence *in situ* hybridization (FISH) analysis of biofilms**

A tissue section from each patient was first stained using a Periodic Acid Schiff (PAS) staining kit (Sigma-Aldrich, USA) to confirm the preservation of the mucus layer according to the manufacturer's directions. Successive sections were then stained with the Eub338 universal bacterial probe for the presence of biofilms (5' - Cy3 - GCTGCCTCCCGTAGGAGT - 3', Sigma-Aldrich, USA)^1,2^ or with the following specific bacterial probes (all from Sigma-Aldrich, USA) for determining the microbial composition of the biofilms: *Fusobacterium* (Fus714; 5' - Cy3 - GGCTTCCCCATCGGCATT - 3')^3^, Bacteroidetes (CFB286; 5' - Alexafluor488 - TCCTCTCAGAACCCCTAC - 3')^4^, Betaproteobacteria (Bet42a; 5' - Cy5 - GCCTTCCCACTTCGTTT - 3')^5^, Gammaproteobacteria (Gam42a; 5' - Cy5 - GCCTTCCCACATCGTTT - 3')^5^, and Lachnospiraceae (Lac435; 5' - TXR - TCTTCCCTGCTGATAGA - 3')^6^. Slides were first deparaffinized with 2 x 5 minute incubations in xylene, then hydrated with 2 x 5 min in 100% ethanol, 2 x 5 min in 95% ethanol, and 5 min in 70% ethanol. Slides were then rinsed in TRIS buffer. Probes were applied to slides at a concentration of 2 μM in a volume of approximately 150 μL per slide in prewarmed hybridization buffer (900 mM NaCl, 20 mM Tris pH 7.5, 0.01% SDS). Slides were incubated at 46°C in a humid chamber for 1.5 hr (universal probe) or 2 hr (multiple probes), then washed 3 x 5 min in wash buffer (215 mM NaCl, 20 mM Tris pH 7.5, 5 mM EDTA). Slides were then counterstained with DAPI (diluted 1:10,000 in PBS) for visualization of nuclei for approximately 5 min at room temperature, then washed again 3 x 5 min with wash buffer. Slide coverslips were mounted with ProLong Gold antifade reagent (Life Technologies). Slides stained with the universal probe were imaged using a Zeiss LSM 510 META laser scanning microscope with LSM Zen imaging software at 400X (including 10X magnification of lens) at the Hopkins Conte Digestive Diseases Basic & Translational Research Core Center. Slides with substantial autofluorescence in the mucus layer and slides stained with multiple probes were imaged on a Zeiss LSM 780 META laser scanning microscope at 400X with LSM Zen imaging software with linear unmixing in the Johns Hopkins University School of Medicine Microscope Core Facility in order to differentiate either autofluorescence from 16S rRNA gene universal probe signal or for differentiation of signal from multiple probes using reference spectra from pure cultures of representative bacteria, respectively.

**Quantification of bacterial biofilms**

Quantification of bacteria was performed using the confocal images above in ImageJ software. A biofilm was defined as having at least 2 x 10^9^ bacteria/mL (equivalent to 1 bacteria per 10 x 10 μm square in a 5 μm thick tissue section), covering an expanse of at least 200 μm long adjacent to the CEC layer^1,7^. A mucosal region adjacent to the CEC layer of at least 200 μm long was drawn for each sample using the line tool in ImageJ. The area of the drawn shape was then measured using the analytical measurement tool, and the number of bacteria within this region were counted manually. Samples with the defined minimum bacterial density above in at least one screened area were designated as biofilm positive. At least three areas were screened per sample.

**16S rRNA gene Illumina library generation and sequencing for MAL1 and MAL2 cohorts**

*MAL1 cohort:* For cell lysis, specimens were resuspended in 700 μL PBS and incubated with lysozyme (5 μL of 10 mg/mL stock), mutanolysin (15 μL of 1 mg/mL stock), and lysostaphin (5 μL of 1 mg/mL stock) in lysing matrix tubes for 30 min at 37°C. A mixture of 10 μL Proteinase K and 50 μL 10% SDS was added, and samples were then vortexed and incubated at 55°C for 45 min. Mechanical lysis was then performed in a FastPrep-24 5G instrument at 6.0 m/s for 40 sec, then centrifuged at 10,000 *g* for 3 min. DNA was then purified using the ZR Fecal DNA MiniPrep kit (Zymo Research). High-throughput next-generation sequencing of the V3-V4 hypervariable region of the 16S rRNA gene was performed using a dual-indexing strategy for multiplexed sequencing described in detail previously^8^. Briefly, PCR reactions were set up in 96-well microtiter plates using the 319F (5'-ACTCCTACGGGAGGCAGCAG-3') and 806R (5'-GGACTACHVGGGTWTCTAAT-3') universal primers, each containing a linker sequence required for Illumina MiSeq 300bp paired-end sequencing, and a 12-bp heterogeneity-spacer index sequence^8,9^. PCR was carried out using the Phusion High-Fidelity DNA polymerase (Thermo Fisher, USA) and 2 ng of template DNA in a 25 μL reaction volume using the following cycling parameters: 30 sec at 98°C, followed by 30 cycles of 10 sec at 98°C, 15 sec at 66°C, and 15 sec at 72°C, with a final step of 10 min at 72°C. Successful amplification was confirmed using gel electrophoresis. Negative controls without DNA template were performed for each primer pair. PCR amplicons were cleaned up and were then normalized using the SequalPrep Normalization Plate kit (Invitrogen Inc., CA, USA) prior to pooling. Sequencing was then performed using the Illumina MiSeq (Illumina, San Diego, CA) according to the manufacturer’s protocol.

*MAL2 cohort:* Genomic DNA was extracted using the MasterPure DNA Purification Kit (Epicentre, Madison, WI, USA) according to the manufacturer’s instructions. The V3-V4 region of the *16S rRNA* gene was amplified using S-D-Bact-0341-b-S-17 forward (5′-NNNNCCTACGGGNGGCWGCAG-3′) and S-D-Bact-0785-a-A-21 reverse (5’-GACTACHVGGGTATCTAATCC-3’) primers^10^ designed to include the Illumina-compatible adaptors as described by Bartram et al^11^. PCR was carried out using NEBNext High-Fidelity Master Mix (New England Biolabs, Ipswich, MA, USA) with the following conditions: initial denaturation at 98°C for 30 sec, followed by 30 cycles consisting of denaturation (98°C for 10 sec), annealing (60°C for 2 min) and extension (72°C for 20 sec) and a final extension step at 72°C for 1 min. Automated cluster generation and a 2 x 250 bp paired-end sequencing was carried out on the MiSeq System (Illumina, San Diego, CA, USA) at the Monash University Malaysia Genomics Facility using the MiSeq 500-cycle reagent kit V2 on standard flow cell.

**Resphera Insight speciation validation and benchmarking studies**

Briefly, Resphera Insight utilizes a manually curated 16S rRNA database with 11,000 unique species, and a hybrid global-local alignment strategy to assign sequences a high-resolution taxonomic lineage^12-14^. This approach attempts to achieve species-level resolution when possible; however, when a confident single species assignment is not feasible, the method minimizes false positives by providing "ambiguous assignments" i.e. a list of candidate species reflecting the ambiguity. For example, if the underlying model is unable to differentiate between *Fusobacterium nucleatum* and *Fusobacterium periodonticum*, Resphera Insight will provide the ambiguous assignment: "*Fusobacterium_nucleatum:Fusobacterium_periodonticum*."

In order to compare the performance of Resphera Insight to other methods for taxonomic assignment, we first obtained high-quality draft genome assemblies of well-defined species from the Human Microbiome Reference Genome Database (<http://hmpdacc.org/HMRGD/>). We extracted full and partial 16S rRNA genes (≥1,000 bp) from a total of 190 species that had been identified with at least 0.5% abundance in any Human Microbiome Project metagenomic sample by the Metaphlan2 benchmark evaluation^15^. For each extracted 16S rRNA gene, we simulated fragments with a 200-500 bp length with a 0.5% error rate from the V3-V4 primer region (5’ of the end of the V3 primer) and submitted those sequences to Resphera Insight, and RDP and UCLUST tools implemented within QIIME (v1.9), that by default are capable of species-level assignment. If a method correctly or incorrectly classified ≥90% of simulated reads to the species level, that species was designated as correctly classified or misclassified, respectively (see Fig. S11). We attempted to compare Mothur (v1.35) and DADA2 (v1.0), but by default these tools do not produce species-level assignments.

To further benchmark the performance of Resphera Insight, we also simulated 200-500 bp reads from the V3-V4 region of reference 16S rRNA genes (5’ of the end of the V3 primer) of 20 *Bacteroides* species and 20 species within the phylum Fusobacteria (Table S3), including members of *Fusobacterium*, *Leptotrichia* and *Cetobacterium*. For each organism, 1,000 sequences were generated, and we introduced a random error rate of 0.5% to reflect the expected base-calling accuracy from the MiSeq platform. Four measures of performance were computed per species (Table S3): True positive rate (TP %) – the percentage of simulated reads with an assignment that includes the correct species (unambiguously or ambiguously); Diagnostic true positive rate (DTP %) – the percentage of simulated reads with an unambiguous assignment to the correct species; False positive rate (FP %) – the percentage of simulated reads with an assignment that did not include the correct species; and False negative rate (FN %) – the percentage of simulated reads that could not be assigned a species-level assignment. These are denoted as “OTUs” by the Insight protocol and are intended to reflect potentially novel species.

**Quantitative real-time PCR**

One hundred nanograms of DNA per reaction from the MAL1 cohort was analyzed by qPCR on an Applied Biosystems 7500 instrument in duplicate for both the *B. fragilis* and *F. nucleatum* 16S rRNA genes using TaqMan Gene Expression Mix and previously published methods^16,17^. The primers and probe for *B. fragilis* 16S rRNA gene were as follows: 16S F - 5'-TCRGGAAGAAAGCTTGCT-3'; 16S R - 5'-CATCCTTTACCGGAATCCT-3'; probe - 5'HEX-AGGGACTGGAAGGCTTTACTGCTTC-3'BHQ1^16^. The primers and probe for *F. nucleatum* 16S rRNA gene were as follows: 16S F - 5'-GGATTTATTGGGCGTAAAGC-3'; 16S R - 5'-GGCATTCCTACAAATATCTACGAA-3'; 16S probe - 5'HEX-TGCAGGGCTCAACTCTGTATTGCG-3'BHQ1. *F. nucleatum* primers were from Boutaga *et al*.^17^; the probe was designed for the present study using the PrimerQuest tool (Integrated DNA Technologies). Cycling parameters were as follows: 50°C 20 min; 95°C 10 min; 40 cycles of 95°C 15 sec, 58°C (*B. fragilis*) or 60°C (*F. nucleatum*) 1 min. Data were fit to a standard curve (10^0^ - 10^7^) using *B. fragilis* strain 086 or a clinical isolate of *F. nucleatum* obtained from Johns Hopkins Hospital.

**Statistical analysis**

Prior to downstream statistical comparisons, 16S rRNA gene profiles within each dataset were subsampled to an even level of coverage. PICRUSt counts associated with functional categories were also normalized to an even total per sample within each dataset. Relative contributions of higher-level taxa to functional categories were determined using the *metagenome_contributions.py* in the PICRUSt package. Diagnostic assignments by Resphera Insight were defined as unambiguous assignments of 16S rRNA gene sequences to the species level. Ambiguous assignments to a species were calculated by aggregating both single species assignments as well as assignments reflecting ties of up to three species in which a particular organism was included. Those results presented here are based on diagnostic assignments unless otherwise noted. Counts were converted to percent abundances and arcsine-square root transformed proportions^18-20^ to evaluate the influence of different normalization methods on our results. Those results presented here are based on percentage abundances unless otherwise noted. Meta-analyses were performed utilizing the *metacont* function in the R package Meta (v. 4.4-1)^21^ employing the Hedges’ *g* standardized mean difference statistic^22^ to calculate fixed and random effects model estimates. The fixed effects model assumes there exists a single effect size shared by all included studies, while the random effects model allows for variation in the effect size from study to study^23^. Heterogeneity analysis includes estimates of *I*^2^ (percentage of variation reflecting true heterogeneity), *τ*^2^ (random-effects between study variance), and p-value from Cochran’s *Q* test^23,24^. To account for potential effect size heterogeneity across studies, we emphasized the random effects model results; those with 95% CI above or below 0 were considered statistically significant. Study heterogeneity was quantified using the *I*^2^ and τ^2^ statistics as well as the *Q* test to assess statistically significant heterogeneity.

**SUPPLEMENTARY REFERENCES**

1 Swidsinski, A., Weber, J., Loening-Baucke, V., Hale, L. P. & Lochs, H. Spatial organization and composition of the mucosal flora in patients with inflammatory bowel disease. *J Clin Microbiol* **43**, 3380-3389, doi:10.1128/JCM.43.7.3380-3389.2005 (2005).

2 Amann, R. I. *et al.* Combination of 16S rRNA-targeted oligonucleotide probes with flow cytometry for analyzing mixed microbial populations. *Appl Environ Microbiol* **56**, 1919-1925 (1990).

3 Valm, A. M. *et al.* Systems-level analysis of microbial community organization through combinatorial labeling and spectral imaging. *Proc Natl Acad Sci U S A* **108**, 4152-4157, doi:10.1073/pnas.1101134108 (2011).

4 Weller, R., Glockner, F. O. & Amann, R. 16S rRNA-targeted oligonucleotide probes for the in situ detection of members of the phylum Cytophaga-Flavobacterium-Bacteroides. *Systematic and applied microbiology* **23**, 107-114, doi:10.1016/S0723-2020(00)80051-X (2000).

5 Manz, W., Amann, R., Ludwig, W., Wagner, M. & Schleifer, K. H. Phylogenetic Oligodeoxynucleotide Probes for the Major Subclasses of Proteobacteria - Problems and Solutions. *Systematic and applied microbiology* **15**, 593-600 (1992).

6 Kong, Y. H., He, M. L., McAlister, T., Seviour, R. & Forster, R. Quantitative Fluorescence In Situ Hybridization of Microbial Communities in the Rumens of Cattle Fed Different Diets. *Appl Environ Microb* **76**, 6933-6938, doi:10.1128/Aem.00217-10 (2010).

7 Dejea, C. M. *et al.* Microbiota organization is a distinct feature of proximal colorectal cancers. *Proc Natl Acad Sci U S A* **111**, 18321-18326, doi:10.1073/pnas.1406199111 (2014).

8 Fadrosh, D. W. *et al.* An improved dual-indexing approach for multiplexed 16S rRNA gene sequencing on the Illumina MiSeq platform. *Microbiome* **2**, 6, doi:10.1186/2049-2618-2-6 (2014).

9 Caporaso, J. G. *et al.* Ultra-high-throughput microbial community analysis on the Illumina HiSeq and MiSeq platforms. *The ISME journal* **6**, 1621-1624, doi:10.1038/ismej.2012.8 (2012).

10 Klindworth, A. *et al.* Evaluation of general 16S ribosomal RNA gene PCR primers for classical and next-generation sequencing-based diversity studies. *Nucleic Acids Res* **41**, e1, doi:10.1093/nar/gks808 (2013).

11 Bartram, A. K., Lynch, M. D., Stearns, J. C., Moreno-Hagelsieb, G. & Neufeld, J. D. Generation of multimillion-sequence 16S rRNA gene libraries from complex microbial communities by assembling paired-end illumina reads. *Appl Environ Microbiol* **77**, 3846-3852, doi:10.1128/AEM.02772-10 (2011).

12 Daquigan, N., Grim, C. J., White, J. R., Hanes, D. E. & Jarvis, K. G. Early Recovery of Salmonella from Food Using a 6-Hour Non-selective Pre-enrichment and Reformulation of Tetrathionate Broth. *Front Microbiol* **7**, 2103, doi:10.3389/fmicb.2016.02103 (2016).

13 Ottesen, A. *et al.* Enrichment dynamics of Listeria monocytogenes and the associated microbiome from naturally contaminated ice cream linked to a listeriosis outbreak. *BMC Microbiol* **16**, 275, doi:10.1186/s12866-016-0894-1 (2016).

14 Abernethy, M. G. *et al.* Urinary Microbiome and Cytokine Levels in Women With Interstitial Cystitis. *Obstet Gynecol* **129**, 500-506, doi:10.1097/AOG.0000000000001892 (2017).

15 Truong, D. T. *et al.* MetaPhlAn2 for enhanced metagenomic taxonomic profiling. *Nat Methods* **12**, 902-903, doi:10.1038/nmeth.3589 (2015).

16 Tong, J., Liu, C., Summanen, P., Xu, H. & Finegold, S. M. Application of quantitative real-time PCR for rapid identification of Bacteroides fragilis group and related organisms in human wound samples. *Anaerobe* **17**, 64-68, doi:10.1016/j.anaerobe.2011.03.004 (2011).

17 Boutaga, K., van Winkelhoff, A. J., Vandenbroucke-Grauls, C. M. & Savelkoul, P. H. Periodontal pathogens: a quantitative comparison of anaerobic culture and real-time PCR. *FEMS immunology and medical microbiology* **45**, 191-199, doi:10.1016/j.femsim.2005.03.011 (2005).

18 Zar, J. H. Biostatistical analysis. 4th. *Upper Saddle River, NJ: Prentice Hall* **1**, 389-394 (1999).

19 Morgan, X. C. *et al.* Dysfunction of the intestinal microbiome in inflammatory bowel disease and treatment. *Genome Biol* **13**, R79, doi:10.1186/gb-2012-13-9-r79 (2012).

20 Liu, Z., Hsiao, W., Cantarel, B. L., Drabek, E. F. & Fraser-Liggett, C. Sparse distance-based learning for simultaneous multiclass classification and feature selection of metagenomic data. *Bioinformatics* **27**, 3242-3249, doi:10.1093/bioinformatics/btr547 (2011).

21 Schwarzer, G. Meta: An R package for meta-analysis. *SpherWave: An R Package for Analyzing Scattered Spherical Data by Spherical Wavelets*, 40 (2007).

22 Hedges, L. V. Distribution theory for Glass's estimator of effect size and related estimators. *Journal of Educational and Behavioral Statistics* **6**, 107-128 (1981).

23 Borenstein, M., Hedges, L. V., Higgins, J. P. & Rothstein, H. R. A basic introduction to fixed-effect and random-effects models for meta-analysis. *Res Synth Methods* **1**, 97-111, doi:10.1002/jrsm.12 (2010).

24 Hoaglin, D. C. Misunderstandings about Q and 'Cochran's Q test' in meta-analysis. *Stat Med* **35**, 485-495, doi:10.1002/sim.6632 (2016).
