# Supplementary material for: High-resolution bacterial 16S rRNA gene profile meta-analysis and biofilm status reveal common colorectal cancer consortia
Source: NPJ Biofilms Microbiomes. 2017 Nov 29;3:34. doi: 10.1038/s41522-017-0040-3 (PMC5707393; doi:10.1038/s41522-017-0040-3)
Supplement: Supplementary file 2 — Fig S1 [file 41522_2017_40_MOESM2_ESM.pdf]

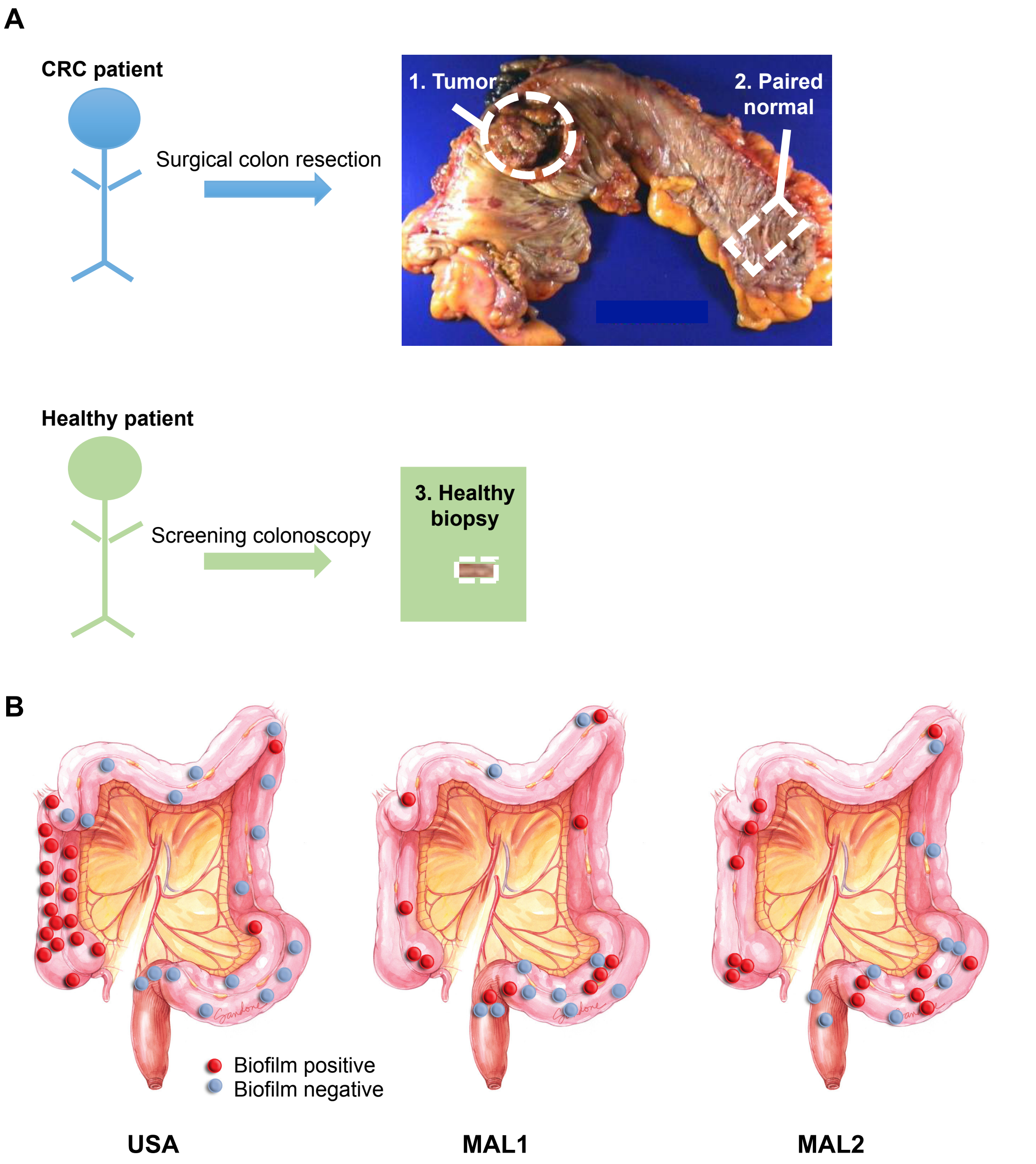

Fig. S1. Colorectal tissue sample collection for biofilm and sequencing studies for USA, MAL1, and MAL2 cohorts. **a** The three major colorectal tissue samples collected from the USA, MAL1, and MAL2 cohorts include (1) CRC tumors harvested during surgical tumor resections from CRC patients, (2) paired, flanking normal tissues harvested during surgical tumor resections from the same CRC patients, and (3) biopsies from grossly

normal colon mucosa (referred to as healthy biopsies) taken during screening colonoscopies from healthy individuals without CRC. **b** Locations of biofilm-positive and biofilm-negative tumors in the USA, MAL1, and MAL2 cohorts. Biofilm-positive samples are colored in red; biofilm-negative samples are colored in blue. The gross colon tumor resection image and the USA biofilm map are reprinted with permission from *PNAS* (Dejea *et al*., 2014). Diagrams are not drawn to scale.
