# Supplementary material for: High-resolution bacterial 16S rRNA gene profile meta-analysis and biofilm status reveal common colorectal cancer consortia
Source: NPJ Biofilms Microbiomes. 2017 Nov 29;3:34. doi: 10.1038/s41522-017-0040-3 (PMC5707393; doi:10.1038/s41522-017-0040-3)
Supplement: Supplementary file 4 — Fig S3 [file 41522_2017_40_MOESM4_ESM.pdf]

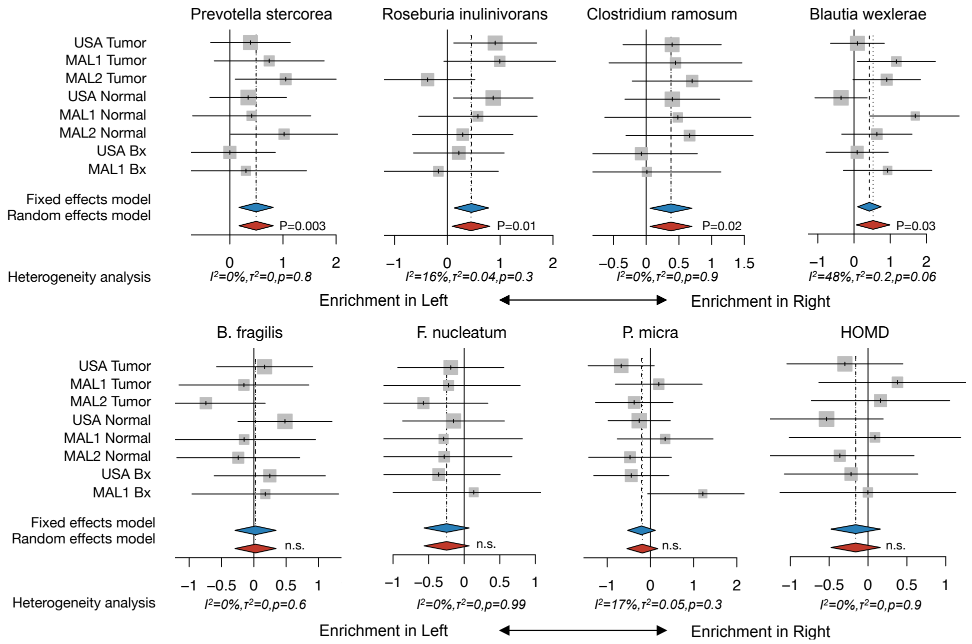

Fig. S3. Differentially abundant species in left- vs. right-sided samples. Frozen tissue from USA, MAL1, and MAL2 cohorts were screened by 16S rRNA gene amplicon sequencing and analyzed using Resphera Insight. Top: several bacterial species were enriched in right-sided samples (proximal to the hepatic flexure) compared to left-sided samples. Bottom: the relative abundances of *B. fragilis*, *F. nucleatum*, *P. micra*, and human oral microbes (HOMD species) were not significantly different between right- and left-sided samples. Random-effects models with 95% CI above or below 0 (red diamonds) were considered statistically significant. Hedge's *g* difference statistic is shown on the X axes. The fixed effects model assumes there exists a single effect size shared by all included studies, while the random effects model allows for variation in the effect size from study to study. Heterogeneity analysis includes estimates of I^2 (percentage of variation reflecting true heterogeneity), τ^2 (random-effects between study variance), and p-value from Cochran's Q test for heterogeneity.
