# Supplementary material for: High-resolution bacterial 16S rRNA gene profile meta-analysis and biofilm status reveal common colorectal cancer consortia
Source: NPJ Biofilms Microbiomes. 2017 Nov 29;3:34. doi: 10.1038/s41522-017-0040-3 (PMC5707393; doi:10.1038/s41522-017-0040-3)
Supplement: Supplementary file 5 — Fig S4 [file 41522_2017_40_MOESM5_ESM.pdf]

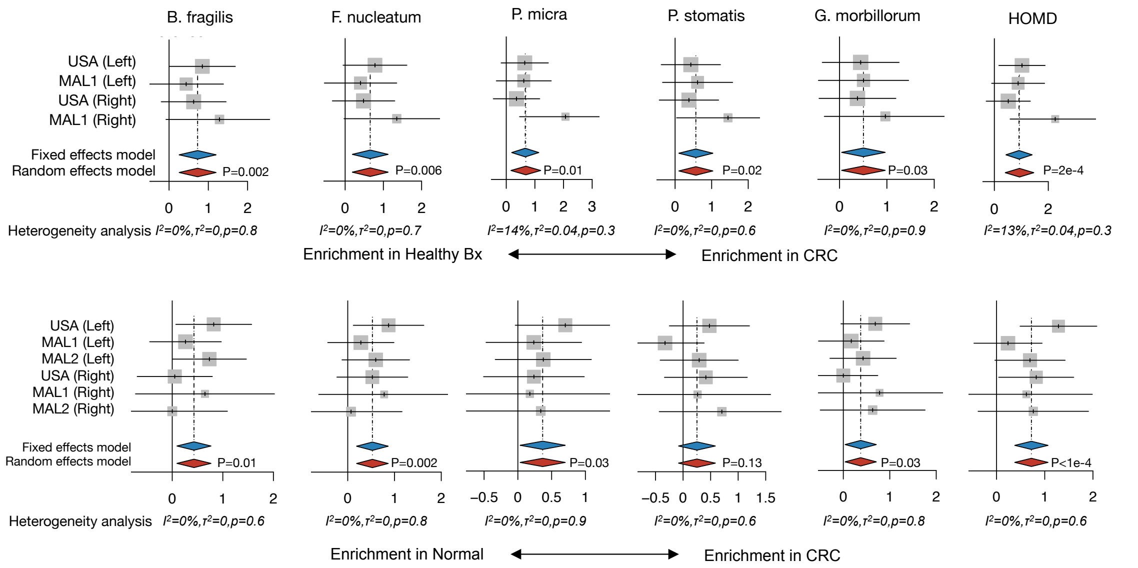

Fig. S4. Associations of *B. fragilis* and human oral microbiota with CRC status after controlling for colon side. Microbiome profiling with 16S rRNA gene amplicon sequencing was applied to tumor (CRC) and paired normal tissues (Normal) from CRC patients for the USA, MAL1, and MAL2 cohorts, as well as healthy biopsies (Healthy Bx) for USA and MAL1. Samples were separated according to their anatomical location (left vs. right). *B. fragilis* as well as the oral pathogens *F. nucleatum*, *P. micra*, *P. stomatis, G. morbillorum*, and the overall consortia associated with the Human Oral Microbiome Database (HOMD) were all found to be significantly enriched in tumor specimens compared to biopsies from healthy patients without cancer (top panel), and compared to normal flanking tissue (bottom panel; with exception of *P. stomatis* in CRC compared to paired normal). Random-effects models with 95% CI above or below 0 (red diamonds) were considered statistically significant. Hedge's *g* difference statistic is shown on the X axes. The fixed effects model assumes there exists a single effect size shared by all included studies, while the random effects model allows for variation in the effect size from study to study. Heterogeneity analysis includes estimates of I^2 (percentage of variation reflecting true heterogeneity), τ^2 (random-effects between study variance), and p-value from Cochran's Q test for heterogeneity.
