# Supplementary material for: High-resolution bacterial 16S rRNA gene profile meta-analysis and biofilm status reveal common colorectal cancer consortia
Source: NPJ Biofilms Microbiomes. 2017 Nov 29;3:34. doi: 10.1038/s41522-017-0040-3 (PMC5707393; doi:10.1038/s41522-017-0040-3)
Supplement: Supplementary file 6 — Fig S5 [file 41522_2017_40_MOESM6_ESM.pdf]

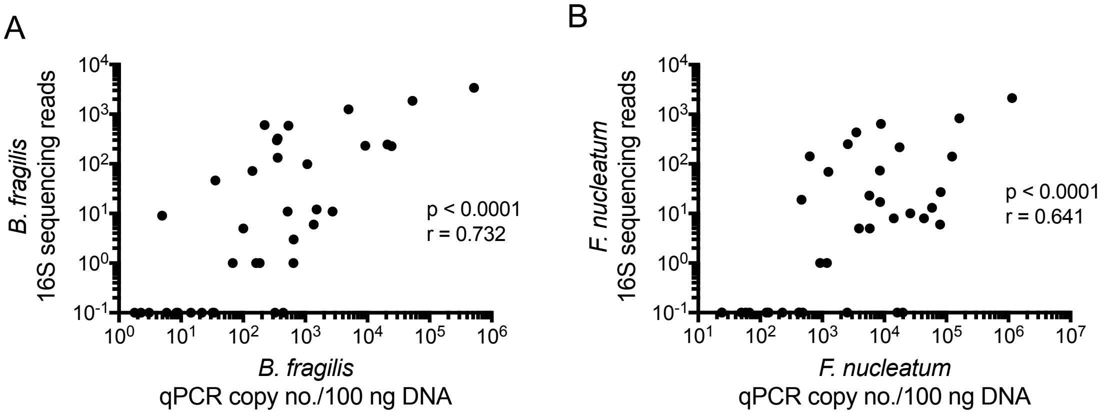

Fig. S5. qPCR validation of Resphera Insight. DNA from the MAL1 cohort was analyzed by qPCR for the 16S rRNA gene for **a** *B. fragilis* and **b** *F. nucleatum*. Samples with 0 reads by 16S rRNA gene sequencing were set to a value of 0.1 for visualization; however, statistical analyses were performed on the original raw data. qPCR data significantly correlated with 16S rRNA gene sequencing data from Resphera Insight using Spearman's rank correlation.
