# Supplementary material for: High-resolution bacterial 16S rRNA gene profile meta-analysis and biofilm status reveal common colorectal cancer consortia
Source: NPJ Biofilms Microbiomes. 2017 Nov 29;3:34. doi: 10.1038/s41522-017-0040-3 (PMC5707393; doi:10.1038/s41522-017-0040-3)
Supplement: Supplementary file 7 — Fig S6 [file 41522_2017_40_MOESM7_ESM.pdf]

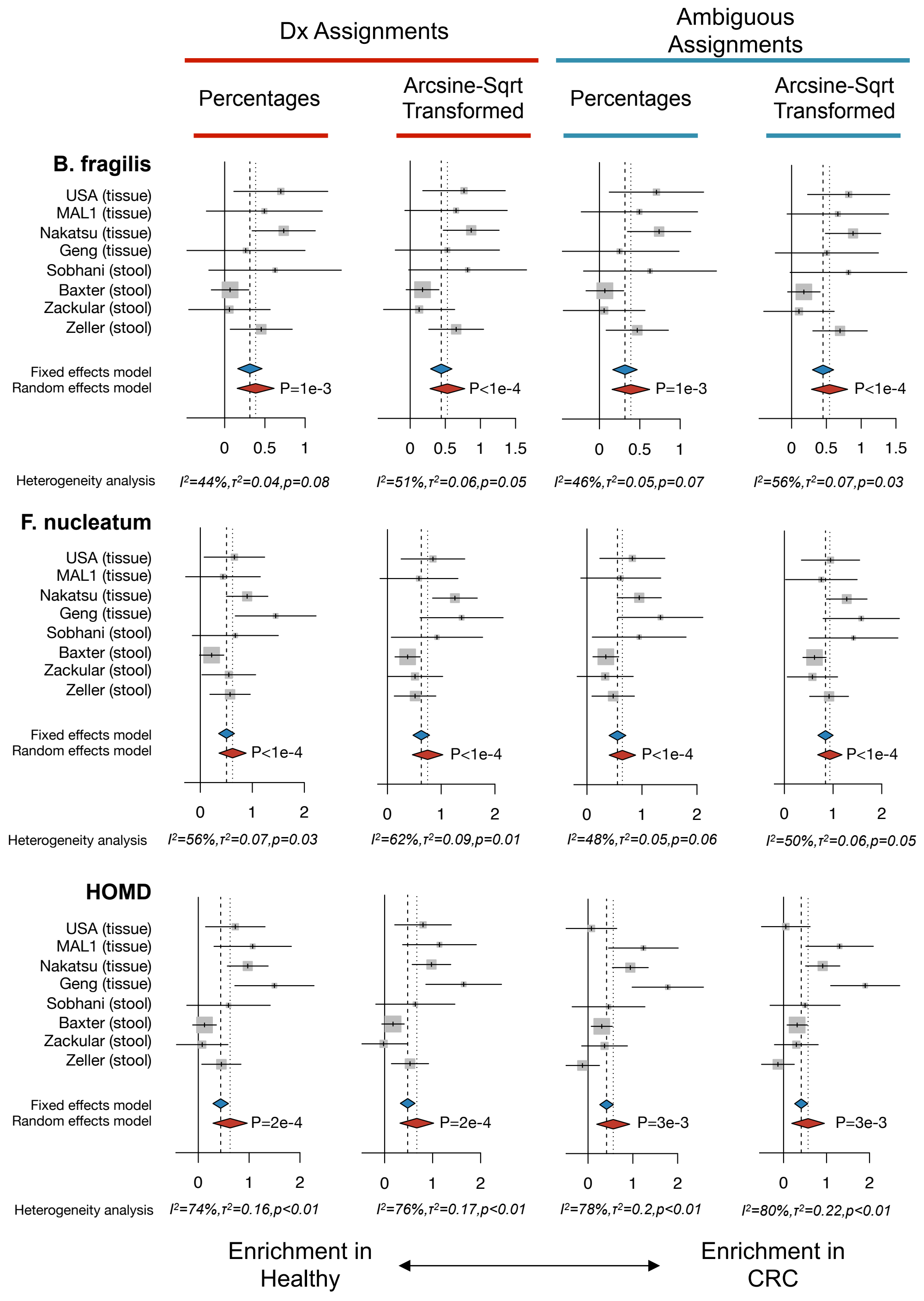

Fig. S6. Robustness of Resphera Insight data analysis. The enrichment of *B. fragilis*, *F. nucleatum*, and HOMD species in healthy tissues vs CRC tissues are demonstrated using various analysis parameters. The statistically significant enrichment of these species are

robust to changes in normalization strategy (i.e., normalization using percentages or arcsine-square-root-transformation) and confidence threshold of assignments (i.e., Diagnostic [Dx] assignments vs. Ambiguous assignments). Random-effects models with 95% CI above or below 0 (red diamonds) were considered statistically significant. Hedge's *g* difference statistic is shown on the X axes. The fixed effects model assumes there exists a single effect size shared by all included studies, while the random effects model allows for variation in the effect size from study to study. Heterogeneity analysis includes estimates of I^2 (percentage of variation reflecting true heterogeneity), τ^2 (random-effects between study variance), and p-value from Cochran's Q test for heterogeneity.
