# Supplementary material for: High-resolution bacterial 16S rRNA gene profile meta-analysis and biofilm status reveal common colorectal cancer consortia
Source: NPJ Biofilms Microbiomes. 2017 Nov 29;3:34. doi: 10.1038/s41522-017-0040-3 (PMC5707393; doi:10.1038/s41522-017-0040-3)
Supplement: Supplementary file 8 — Fig S7 [file 41522_2017_40_MOESM8_ESM.pdf]

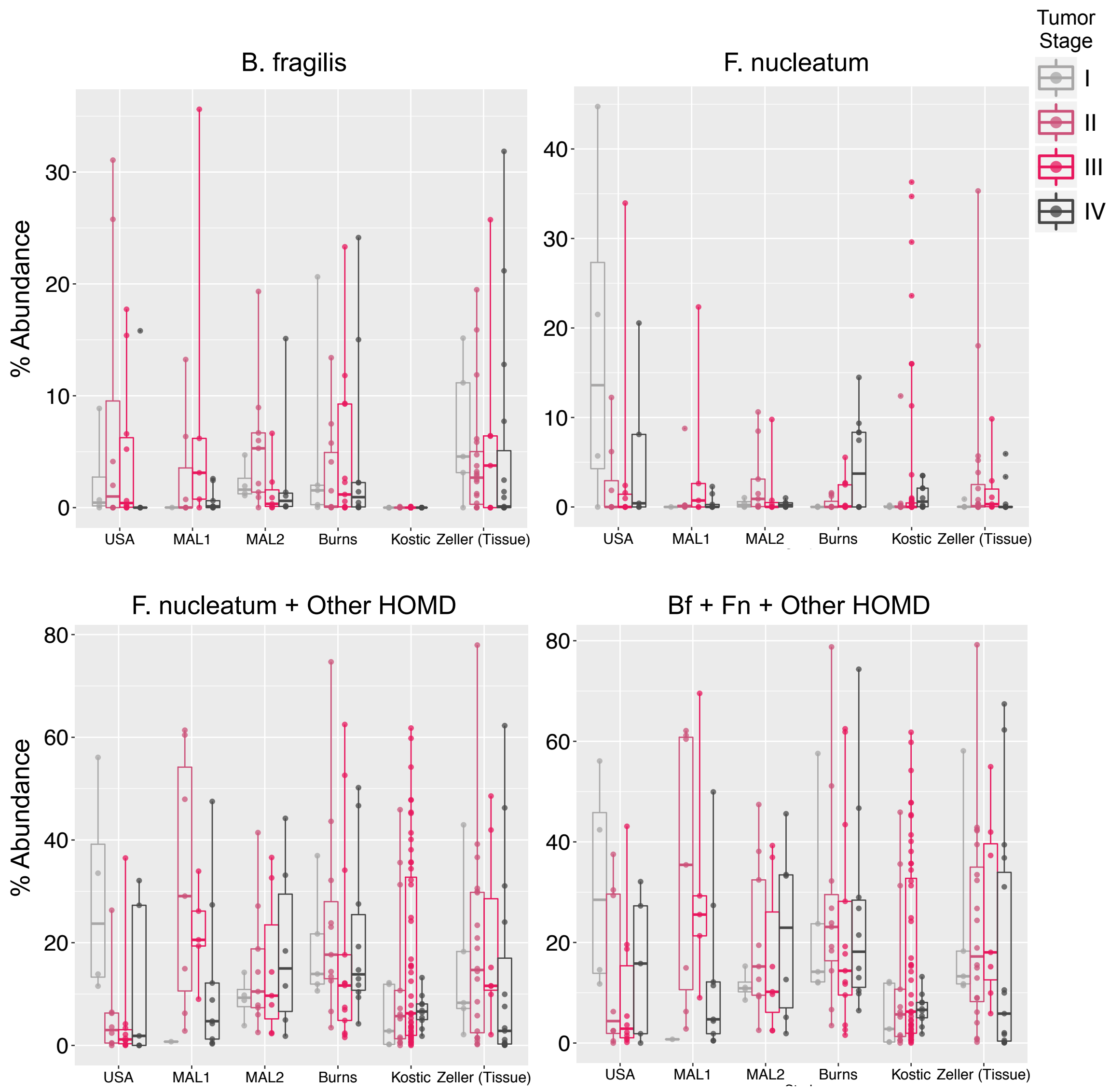

Fig. S7. Associations of specific species with clinical tumor stage. The percent abundance of sequences from *B. fragilis* (top left), *F. nucleatum* (top right), *F. nucleatum* + other HOMD (bottom left), and *B. fragilis*, *F. nucleatum*, + other HOMD (bottom right) compared to the total number of sequences from 16S rRNA gene sequencing are presented according to clinical tumor stage status for studies in which tumor stage information was available.
