# Supplementary material for: High-resolution bacterial 16S rRNA gene profile meta-analysis and biofilm status reveal common colorectal cancer consortia
Source: NPJ Biofilms Microbiomes. 2017 Nov 29;3:34. doi: 10.1038/s41522-017-0040-3 (PMC5707393; doi:10.1038/s41522-017-0040-3)
Supplement: Supplementary file 9 — Fig S8 [file 41522_2017_40_MOESM9_ESM.pdf]

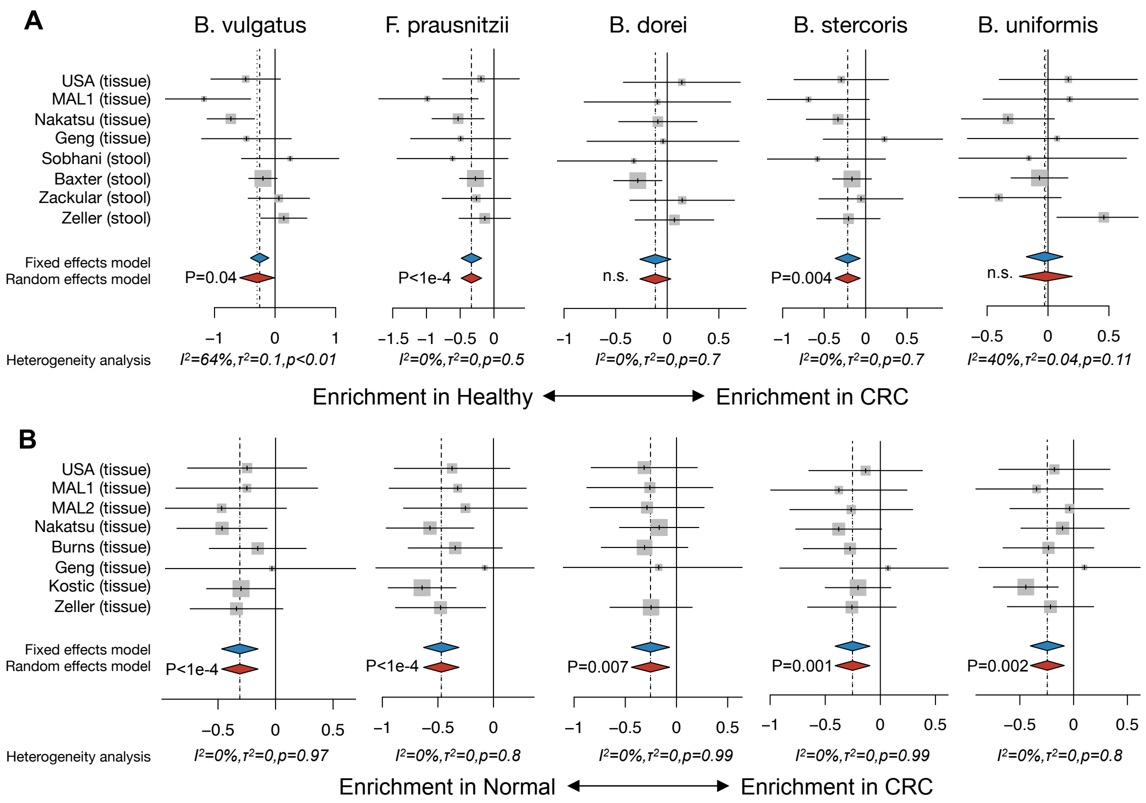

Fig. S8. Additional changes in species abundance in the expanded meta-analysis. Several *Bacteroides* species (*B.vulgatus*, *B. dorei*, and *B. stercoris*) as well as *Faecalibacterium prausnitzii* were consistently depleted in CRC compared to **a** healthy biopsies and **b** paired normal tissues using Resphera Insight analysis of 16S rRNA gene sequencing data. Random-effects models with 95% CI above or below 0 (red diamonds) were considered statistically significant. Hedge's *g* difference statistic is shown on the X axes. The fixed effects model assumes there exists a single effect size shared by all included studies, while the random effects model allows for variation in the effect size from study to study. Heterogeneity analysis includes estimates of I^2 (percentage of variation reflecting true heterogeneity), τ^2 (random-effects between study variance), and p-value from Cochran's *Q* test for heterogeneity.
