# Supplementary material for: High-resolution bacterial 16S rRNA gene profile meta-analysis and biofilm status reveal common colorectal cancer consortia
Source: NPJ Biofilms Microbiomes. 2017 Nov 29;3:34. doi: 10.1038/s41522-017-0040-3 (PMC5707393; doi:10.1038/s41522-017-0040-3)
Supplement: Supplementary file 10 — Fig S9 [file 41522_2017_40_MOESM10_ESM.pdf]

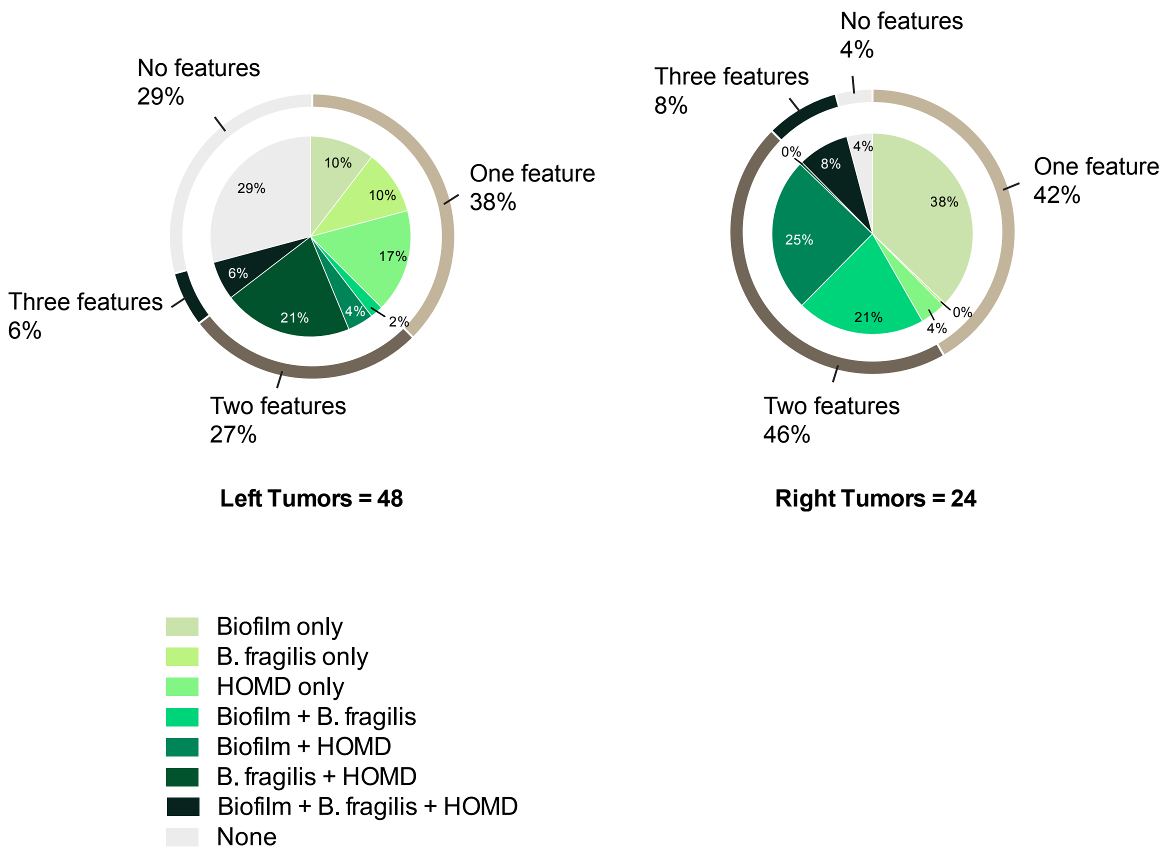

Fig. S9. Prevalence of microbial features in left- vs right-sided tumors from the USA, MAL1, and MAL2 cohorts. All tumors from the three cohorts for which data on biofilms and 16S rRNA gene sequencing were available were included in this analysis. Positive status for *B. fragilis* was defined as an abundance >2% of all microbial sequences, while positive status for human oral microbes from the HOMD database were defined as an abundance >10% of all microbial sequences from the 16S rRNA gene analysis. Data are presented according to tumor side (left panel, left-sided CRC; right panel, right-sided CRC).
