# Supplementary material for: High-resolution bacterial 16S rRNA gene profile meta-analysis and biofilm status reveal common colorectal cancer consortia
Source: NPJ Biofilms Microbiomes. 2017 Nov 29;3:34. doi: 10.1038/s41522-017-0040-3 (PMC5707393; doi:10.1038/s41522-017-0040-3)
Supplement: Supplementary file 11 — Fig S10 [file 41522_2017_40_MOESM11_ESM.pdf]

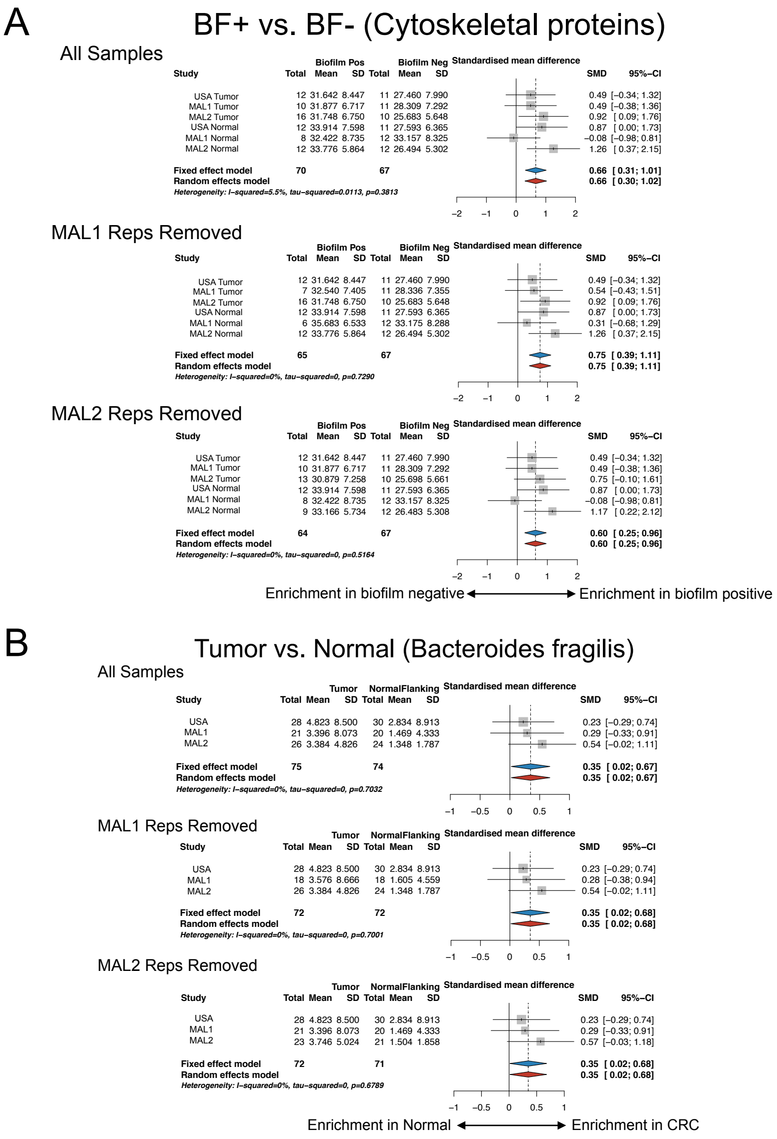

Fig. S10. Resphera Insight analysis with and without biological replicates. Representative plots of the Resphera Insight analysis of the **a** PICRUSt analysis of the 16S rRNA gene sequencing data from biofilm positive vs biofilm negative samples and **b** tumor vs paired normal samples for USA, MAL1, and MAL2. The 3 biological replicates were either included (top panel), removed from MAL1 (middle panel), or removed from MAL2 (bottom panel). The fixed effects model assumes there exists a single effect size shared by all included studies, while the random effects model allows for variation in the effect size from study to study. Heterogeneity analysis includes estimates of I^2 (percentage of variation reflecting true heterogeneity), τ^2 (random-effects between study variance), and p-value from Cochran's Q test for heterogeneity.
