# Supplementary material for: High-resolution bacterial 16S rRNA gene profile meta-analysis and biofilm status reveal common colorectal cancer consortia
Source: NPJ Biofilms Microbiomes. 2017 Nov 29;3:34. doi: 10.1038/s41522-017-0040-3 (PMC5707393; doi:10.1038/s41522-017-0040-3)
Supplement: Supplementary file 12 — Fig S11 [file 41522_2017_40_MOESM12_ESM.pdf]

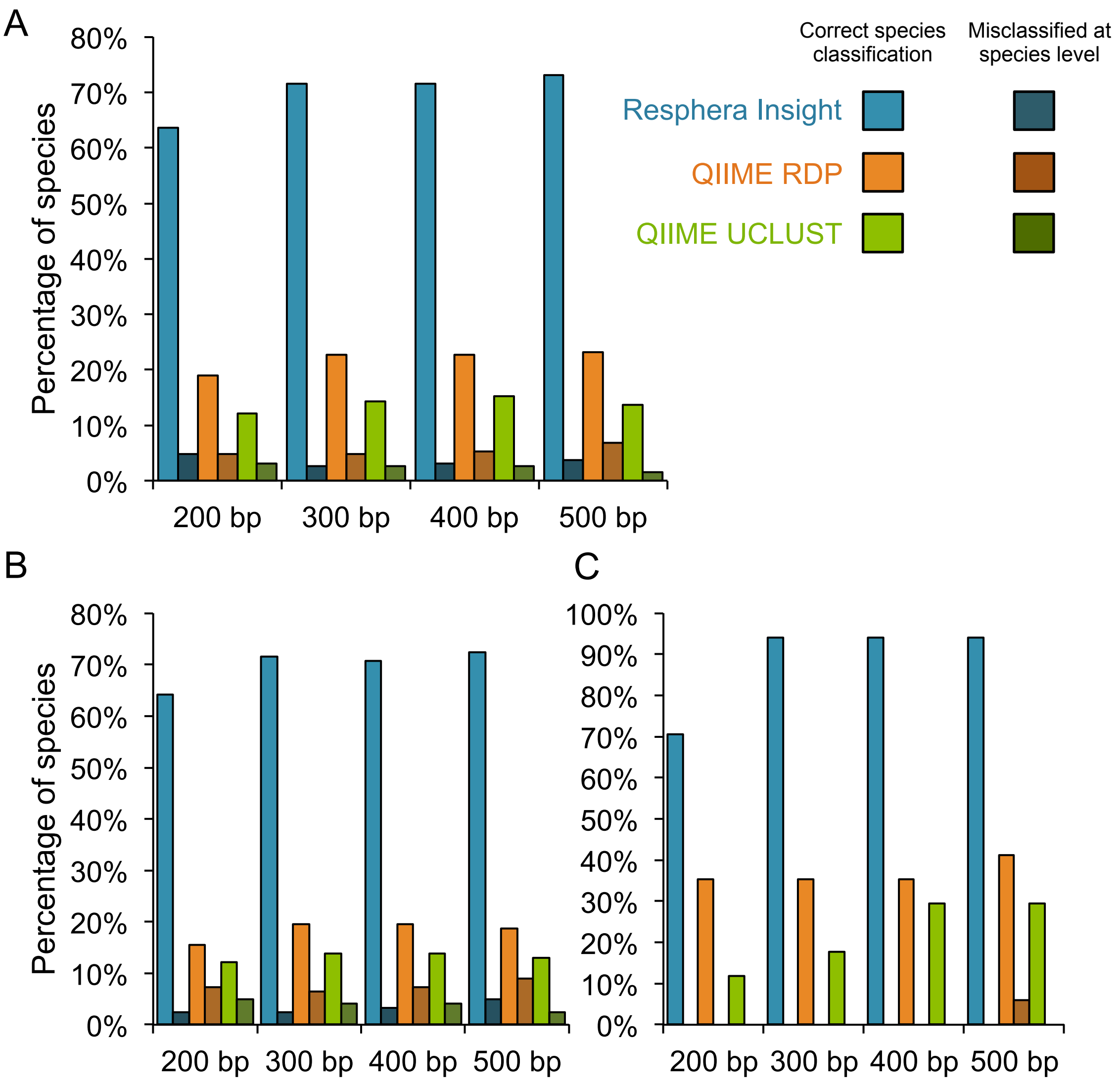

Fig. S11. Sensitivity and specificity of Resphera Insight on the HMP reference isolate collection. *In silico* sensitivity and mis-assignment rates of Resphera Insight compared to QIIME RDP and QIIME UCLUST for **a** a collection of representative species from the HMP reference genome set (N = 190), **b** species listed by Human Oral Microbiome Database (N = 123), and **c** species within the genus Bacteroides (N = 17). See Methods for validation description.
